# CRISPR/Cas9 Targeted Editing of Genes Associated With Fungal Susceptibility in *Vitis vinifera* L. cv. Thompson Seedless Using Geminivirus-Derived Replicons

**DOI:** 10.3389/fpls.2021.791030

**Published:** 2021-12-23

**Authors:** Felipe Olivares, Rodrigo Loyola, Blanca Olmedo, María de los Ángeles Miccono, Carlos Aguirre, Ricardo Vergara, Danae Riquelme, Gabriela Madrid, Philippe Plantat, Roxana Mora, Daniel Espinoza, Humberto Prieto

**Affiliations:** ^1^Biotechnology Laboratory, La Platina Research Station, National Institute of Agriculture Research, Santiago, Chile; ^2^Phytopathology Laboratory, La Platina Research Station, National Institute of Agriculture Research, Santiago, Chile

**Keywords:** grapevine gene editing, BeYDV-derived vector, *Agrobacterium*-mediated transformation, paired gRNA gene editing, fungal susceptibility genes

## Abstract

The woody nature of grapevine (*Vitis vinifera* L.) has hindered the development of efficient gene editing strategies to improve this species. The lack of highly efficient gene transfer techniques, which, furthermore, are applied in multicellular explants such as somatic embryos, are additional technical handicaps to gene editing in the vine. The inclusion of geminivirus-based replicons in regular T-DNA vectors can enhance the expression of clustered regularly interspaced short palindromic repeats/CRISPR-associated protein 9 (CRISPR/Cas9) elements, thus enabling the use of these multicellular explants as starting materials. In this study, we used *Bean yellow dwarf virus* (BeYDV)-derived replicon vectors to express the key components of CRISPR/Cas9 system *in vivo* and evaluate their editing capability in individuals derived from *Agrobacterium*-mediated gene transfer experiments of ‘Thompson Seedless’ somatic embryos. Preliminary assays using a BeYDV-derived vector for *green fluorescent protein* reporter gene expression demonstrated marker visualization in embryos for up to 33 days post-infiltration. A universal BeYDV-based vector (pGMV-U) was assembled to produce all CRISPR/Cas9 components with up to four independent guide RNA (gRNA) expression cassettes. With a focus on fungal tolerance, we used gRNA pairs to address considerably large deletions of putative grape susceptibility genes, including *AUXIN INDUCED IN ROOT CULTURE 12* (*VviAIR12*), *SUGARS WILL EVENTUALLY BE EXPORTED TRANSPORTER 4* (*VviSWEET4*), *LESION INITIATION 2* (*VviLIN2*), and *DIMERIZATION PARTNER-E2F-LIKE 1* (*VviDEL1*). The editing functionality of gRNA pairs in pGMV-U was evaluated by grapevine leaf agroinfiltration assays, thus enabling longer-term embryo transformations. These experiments allowed for the establishment of greenhouse individuals exhibiting a double-cut edited status for all targeted genes under different allele-editing conditions. After approximately 18 months, the edited grapevine plants were preliminary evaluated regarding its resistance to *Erysiphe necator* and *Botrytis cinerea*. Assays have shown that a transgene-free *VviDEL1* double-cut edited line exhibits over 90% reduction in symptoms triggered by powdery mildew infection. These results point to the use of geminivirus-based replicons for gene editing in grapevine and other relevant fruit species.

## Introduction

Grape (*Vitis vinifera* L.) is a perennial fruit crop with regionally high economic activity due to its multiproduct nature (Moriondo et al., 2011). On a productive scale, the management of fungal diseases represents a major challenge in viticulture. Containing the growth of pathogens and the progress of plant disease depend on chemicals, with application regimes that even include preventive treatments. Therefore, solutions with a trend toward a sustainable and agrochemical-free agriculture and production chain are needed.

Genomic studies and technological advances in plant genetic engineering provide a path for developing new varieties compatible with today’s market and production challenges (Gomès et al., 2021). In this regard, strategies for genetically improving grapevine rely on both conventional and precision breeding. The latter involves gene editing (GenEd) techniques actively under development (Zhu et al., 2020). Complete sequencing of the grapevine genome (Jaillon et al., 2007) has identified novel genes, analyzed structural gene variants, discovered new single nucleotide polymorphisms, and clarified regulatory regions. These insights enable precision breeding using GenEd tools (Capriotti et al., 2020; Paul et al., 2021). The clustered regularly interspaced short palindromic repeats/CRISPR-associated protein 9 (CRISPR/Cas9) system is currently one of the most powerful GenEd techniques available. It allows the direct generation of target-specific sequence modifications in the genome, opening DNA repair pathways via donor-dependent homology-directed recombination or error-prone non-homologous end joining, activated after the Cas9-induced DNA double-stranded break (DSB). Target-specific sequence recognition by Cas9 is led by guide RNAs (gRNAs) (van der Oost et al., 2014; Jiang and Doudna, 2017), a short synthetic RNA fragment composed of a scaffold sequence necessary for Cas-binding, and a user-defined spacer of ∼20 nucleotides based on the genomic target to be modified (Sternberg et al., 2014). Finally, if the identified target sequence is contiguous to a 3-bp protospacer-adjacent motif (PAM), Cas9 will generate a DSB, enabling the indicated DNA repair pathways and, in that way, target-specific sequence modifications (Hille and Charpentier, 2016; Jiang and Doudna, 2017).

In grapes, CRISPR/Cas9 GenEd has been reported since 2016 (Ren et al., 2016), primarily using binary Ti-derived plasmids suitable for *Agrobacterium-*mediated gene transfer for expressing gRNAs and Cas9 mRNA. Ren et al. (2016) transformed ‘Chardonnay’ embryogenic cell masses, which led to point mutations in the *L-IDONATE DEHYDROGENASE* (*L-IdnDH*) gene in cell lines and regenerated whole plants. In addition, ‘Neo Muscat’ somatic embryos were transformed with editing constructs targeting the *PHYTOENE DESATURASE* (*PDS*) gene, which in turn generated whole plants with albino leaves (Nakajima et al., 2017). With a focus on phytopathology, transgenic ‘Thompson Seedless’ plants were also recently produced with mutated versions of the *WRKY52* transcription factor gene under both mono- and bi-allelic conditions leading to regenerated plants with increased tolerance to *Botrytis cinerea*, the agent causing gray mold disease (Wang et al., 2018). More recently, Li et al. (2020) described using pro-embryogenic cells for gene transfer experiments to generate ‘Thompson Seedless’ with a loss of function of the *PATHOGENESIS-RELATED* 4b (*pr4b*) gene, underscoring the importance of this gene in downy mildew susceptibility. Advances in grapevine GenEd that avoid transgenic conditions have emerged with the aim of producing individuals with improved tolerance to the global problem of powdery mildew caused by the biotrophic *Erysiphe necator*. Direct delivery of purified Cas9 and gRNAs to protoplasts targeting the *MILDEW RESISTANCE LOCUS O 7* (*VviMLO7*) gene in ‘Chardonnay’ (Malnoy et al., 2016) and the *DOWNY MILDEW RESISTANCE 6-2* (*VviDMR6-2*) susceptibility gene in ‘Crimson Seedless’ (Scintilla et al., 2021) has shown the complexity of successful protoplast regeneration in the species, but only the latter described the production of edited whole plants.

Motivated by the lack of efficient regeneration protocols for many commercially relevant plant species, progress has been made by adapting the use of *Agrobacterium*-mediated gene transfer procedures supported by regular regeneration protocols (Oh et al., 2021; Vergara et al., 2021). Among these tools, T-DNA-derived vectors harboring key elements from the autonomously replicating geminivirus genome (i.e., geminivirus-derived replicons), have been demonstrated to improve the delivery of CRISPR/Cas9 components into plant cells and successfully achieve GenEd (Baltes et al., 2014; Čermák et al., 2015; Butler et al., 2016; Acha et al., 2021). In addition, the use of single gRNAs to induce nucleotide-level GenEd (mainly short deletions and/or insertions) and eventual loss of activity of specific genes due to the DSB generated by Cas9, can be improved via a double gRNA approach to mediate larger DNA fragment deletions. Several studies have demonstrated that CRISPR/Cas9-mediated large deletions can be successfully produced via a paired gRNA strategy (Zhou H. et al., 2014; Zhao et al., 2016; Kapusi et al., 2017; Wang et al., 2017; Cai et al., 2018; Wu et al., 2018; Li et al., 2019; Liu et al., 2020; Acha et al., 2021; Duan et al., 2021). However, the use of geminivirus-replicons for GenEd and the generation of larger DNA deletions to increase the chance of gene inactivation have not been explored in grapevines.

Here, we employed CRISPR/Cas9 technology combined with the paired gRNA approach to specifically induce large deletions in several susceptibility genes related to fungus-grapevine interaction through a geminivirus-derived vector. Four putative grape homolog genes were selected based on previous studies in *Arabidopsis thaliana*. These are individual targets for CRISPR/Cas9-mediated gene deletions: (A) the *AUXIN INDUCED IN ROOT CULTURE 12* (*AIR12*) gene, which plays a role in the regulation of the apoplast redox state and the response of the plant to abiotic and biotic stress, promoting pathogen infection (Costa et al., 2015). (B) The *SUGARS WILL EVENTUALLY BE EXPORTED TRANSPORTER 4* (*SWEET4*) gene is a member of a developmentally regulated sugar transporter gene family that has been described as “hijacked” by pathogens to sustain their growth (Baker et al., 2012; Chong et al., 2014). (C) The *LESION INITIATION 2* (*LIN2*) gene encodes a coproporphyrinogen III oxidase, a key enzyme in the tetrapyrrole biosynthesis pathway (Guo et al., 2013) whose mutants induce the initiation step in the formation of limited lesions that mimic a hypersensitivity response and limit pathogen infection. (D) The *DIMERIZATION PARTNER-E2F-LIKE 1* (*DEL1*) gene is a transcriptional repressor known to promote cell proliferation and acts as a negative regulator of salicylic acid (SA) accumulation and plant defense (Wildermuth, 2010; Chandran et al., 2014). The deletion of large DNA fragments for all targeted genes was observed in the regenerated plants from GenEd experiments, which were established and raised in a greenhouse for 1 year. These results are presented and discussed while stressing technical considerations to improve the use of geminivirus-based replicons in grapevine GenEd.

## Materials and Methods

### Plant Material, Somatic Embryogenesis, and Gene Transfer

Certified virus-free ‘Thompson Seedless’ plants have been established under *in vitro* culture (Rubio et al., 2015) and propagated in 0.8x MS (Murashige and Skoog, 1962) medium. Multiple budding was induced in the plantlets using C2D medium (Chée and Pool, 1987) supplemented with 6-benzyladenine (4 μM). New buds were propagated during a 30-day regime. Cultivation chambers for these materials were set at 24 ± 2°C with a constant photoperiod of 16 h light/8 h darkness. For somatic embryogenesis, apical and axial buds with 2–4 leaves were cut from *in vitro*-grown plants. Buds were excised using a stereoscopic lens and incubated for callus induction in NB2 medium (Nitsch and Nitsch, 1969) following the general procedures introduced by Li et al. (2001) by cultivation in darkness for 40–90 days at 24 ± 2°C to form pro-embryogenic calli. Pro-embryogenic and embryogenic masses were transferred to X6 medium and kept at the same temperature under a 16 h light/8 h darkness photoperiod for a propagation phase of up to 90 days. The media were refreshed every 45 days. For gene transfer experiments, approximately 200 mg of embryogenic masses were transferred to solid DM medium (Driver and Kuniyuki, 1984) for a 7–10 days pre-culture treatment before co-cultivation with *Agrobacterium*. Inoculation and co-cultivation procedures were carried out as described by Tapia et al. (2009). After co-cultivation, embryogenic callus cells were washed with water, transferred to DMcc200 medium (DM supplemented with cefotaxime 200 mg/L, carbenicillin 200 mg/L), and cultivated for 21 days. Subsequently, callus cells were transferred to X6cc200 (X6 supplemented with cefotaxime 200 mg/L, carbenicillin 200 mg/L) to induce embryo formation within a period of 4–6 months. Cultures were refreshed every 45 days. New embryo masses were selected and transferred to liquid G11 medium [Ca(NO_3_)_2_*4H_2_O (355 mg/L), Na_2_EDTA (37.3 mg/L), FeSO_4_*7H_2_O (27.8 mg/L), H_2_BO_3_ (6.2 mg/L), Na_2_MoO_4_*2H_2_O (0.25 mg/L), CoCl_2_*6H_2_O (0.025 mg/L), KH_2_PO_4_ (204 mg/L), MgSO_4_*7H_2_O (370 mg/L), MnSO_4_*H_2_O (0.85 mg/L), ZnSO_4_*7H_2_O (8.6 mg/L), CuSO_4_*5H_2_O (0.025 mg/L), KI (0.83 mg/L), nicotinic acid (1 mg/L), pyridoxine-HCl (2 mg/L), thiamine-HCl (3 mg/L), KNO_3_ (950 mg/L), NH_4_NO_3_ (825 mg/L), glycine (2 mg/L), indole butyric acid (0.05 mg/L), benzyl aminopurine (0.5 mg/L), myo-inositol (100 mg/L), sucrose (30 g/L), activated charcoal (0.2 g/L), pH 5.8] for embryo germination (usually 15 days). Germinated embryos were transferred to C2D medium for plantlet establishment, a process requiring approximately 60 days (in which plants achieved an approximate height of 10 cm). The plantlets were propagated every 45 days.

### Plasmids

We built a universal Geminivirus vector (pGMV-U, Addgene plasmid # 112797) by the PCR amplification of fragments from the BeYDV-based LSL vector pTC223 (Addgene plasmid #70019; Čermák et al., 2015) and the T-DNA binary vector pHSE401 (Addgene plasmid #62201; Xing et al., 2014). The characterization of the BeYDV replicon’s behavior in grapevine was performed with the pLSLGFP.R vector (Addgene plasmid # 51501; Baltes et al., 2014) whose T-DNA contains a BeYDV-derived replicon with a green fluorescent protein (GFP) expression cassette. The vectors pLSLGFP.R (Addgene plasmid # 51501) and pTC223 (Addgene plasmid #70019) were a kind gift from Dr. Daniel Voytas (University of Minnesota) and vector pHSE401 was a gift from Qi-Jun Chen (Addgene plasmid # 62201).

### Time-Course Analysis of the Bean Yellow Dwarf Virus-Derived Replicon in Grapevine Tissues

**FIGURE 1:**
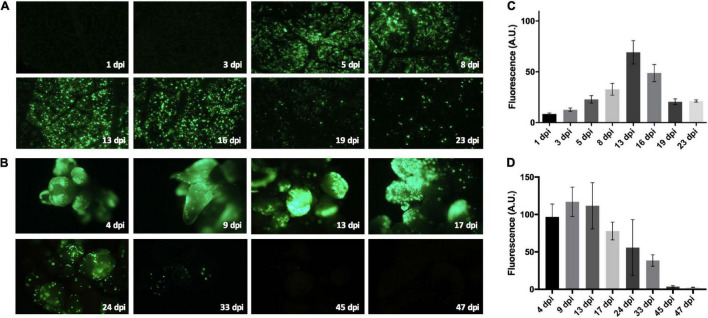
Bean yellow dwarf virus (BeYDV)-derived vector behavior in ‘Thompson Seedless’ grapevine. An LSL vector based on the BeYDV and harboring a green fluorescent protein (*GFP*) expression cassette was used in gene transfer experiments in leaves **(A)** and somatic embryos **(B)**. Both explants were evaluated at different days post-infiltration (dpi) using an epifluorescence microscope to acquire digital images for each time point. Eight images per dpi time point were processed using ImageJ software to quantify the light intensity per pixel in the green channel. Leaf images were evaluated from vacuum agroinfiltrations of whole plants. The mean light intensities for the leaves **(C)** and somatic embryos **(D)** were calculated to produce intensity plots with the means represented as arbitrary units (A.U.) of fluorescence per pixel. The mean intensities were subjected to one-way ANOVA. Three full-length experiments were performed. Standard error bars are shown.

Evaluation of GFP expression over time was achieved through the use of whole plant vacuum-agroinfiltration assays (as described by Chialva et al., 2018) along with gene transfer experiments in ‘Thompson Seedless’ somatic embryos. The *GFP* reporter gene expression was evaluated by epifluorescence microscopy in leaves at 1, 3, 5, 8, 13, 16, 19, and 23 days post-infiltration (dpi) (Figure 1A) and in embryogenic callus between 4 and 47 dpi (Figure 1B). Samples were observed using a Zeiss Axioscope Lab A.1 epifluorescence microscope equipped with Filter Set 09 (BP 450–490 nm) and Filter Set 38 (BP 470/540 nm; Zeiss, Oberkochen, Germany). The light source was a 470-nm LED lamp. Images were acquired with a Canon Rebel T3 camera using EOS Utility software (Canon Inc., Tokyo, Japan). The green channel was quantified via eight images per point using ImageJ software (National Institutes of Health, United States). Data was subjected to one-way ANOVA test with a 5% level of significance using Statgraphics Centurion XV (Manugistics Inc., Rockville, MD, United States).

### Universal Geminivirus Vector Design and Construction

Four PCR fragments with overlapping ends were concatenated using Gibson assembly (Gibson, 2011). The primers used for these amplifications (Supplementary Table S1) were designed by Snapgene software (from GSL Biotech^1^). The PCR reactions were performed using Kapa HiFi DNA Polymerase (Kapa Biosystems, Wilmington, MA, United States) according to the manufacturer instructions. PCR products were resolved by electrophoresis in 1% agarose gels, stained with ethidium bromide (EtBr), and extracted using the Zymoclean Gel DNA Recovery Kit (Zymo Research, Irvine, CA, United States). A total of 100 ng per purified fragment was utilized for the assembly reaction. The Gibson reaction was performed with an assembly master mix (New England BioLabs, Ipswich, MA, United States) following the manufacturer’s instructions. The resulting vector was cloned into *Escherichia coli* Top 10 competent cells (Thermo Fisher Scientific, Waltham, MA, United States). The assembled regions in the pGMV-U plasmid were amplified by PCR using the primers described in Supplementary Table S1 and sequenced for validation (Macrogen Inc., Seoul, South Korea).

### Development of a CRISPR Guide RNA Search Tool for Grapevine

We implemented a dedicated tool to process genome information for grapevine that generated gRNA pairs for efficient GenEd (hereafter “Grapevine CRISPR Search Tool”). The system was based on CRISPR-Analyzer (Shen et al., 2014), CRISPETa (Pulido-Quetglas et al., 2017) and the “Potato CRISPR Search Tool” detailed in Acha et al. (2021). Databases were built based on the *V. vinifera* reference genome (Genoscope 12X; Jaillon et al., 2007). The tool allows the resulting gRNA pairs to be examined for off-targets across the entire grape genome. The off-target results are individualized according to chromosome, sequence coordinates, mismatch number and position, and location (exonic, intronic, or intergenic).

### Guide RNA Pairs Design for the Target Genes and Cloning in Universal Geminivirus Vector

Guide gRNA pairs were generated using the “Grapevine CRISPR Search Tool” described above. The putative sequences and locations of the target genes were found in both the grapevine reference genome (‘Pinot Noir’; Jaillon et al., 2007) and ‘Thompson Seedless’ genomic datasets (Di Genova et al., 2014), and later they were experimentally cloned, sequenced and submitted to GenBank^®2^ (*VviAIR12*, GenBank accession no. MZ031988; *VviSWEET4*, GenBank accession no. MZ031989; *VviLIN2*, GenBank accession no. MZ031990; *VviDEL1*, GenBank accession no. MZ031991) (Table 1). The sequences of the target genes were first compared by pairwise sequence alignment using a CLC Genomics Workbench (Qiagen, Redwood City, CA, United States). The conserved regions resulting from this alignment were used for the gRNA search and specific studies (i.e., *SWEET* genes) were considered for complex gene families (Chong et al., 2014). The guide RNA criteria during selection with the tool were as follows: no off-targets with two or fewer mismatches, maximum of three off-targets with three mismatches and 30 off-targets with four mismatches, and minimum individual and paired scores of 0.2 and 0.4, respectively (Supplementary Table S2). The selected gRNA pairs (gRNA1 + gRNA2 for each gene) were cloned into pGMV-U through a Golden Gate reaction as described by Xing et al. (2014) with the following modifications: The PCR reaction was performed using Platinum SuperFi proofreading DNA polymerase (Thermo Fisher Scientific) according to the manufacturer’s specifications. Primers DT1 and DT2 for each gene were incorporated into a PCR reaction with a final volume of 50 μL (Supplementary Table S1; nomenclature according to Xing et al., 2014). The PCR product was then electrophoresed and purified from EtBr-stained 1.2% agarose gels with the ZymoClean Gel Recovery kit (Zymo Research) and eluted with 6 μL of nuclease-free water. The Golden Gate reaction was performed with the following conditions: 30 cycles of digestion/ligation at 37°C for 10 min and 16°C for 10 min, respectively; a final digestion at 55°C for 10 min; and a denaturation round at 80°C for 15 min. The ligated pGMV-X vector (in which “X” represents the target gene *VviAIR12*, *VviSWEET4, VviLIN2*, or *VviDEL1*) was transformed into *E. coli* Top 10 competent cells (Thermo Fisher Scientific) and selected with 50 μg/mL kanamycin following the manufacturer’s instructions. The recombinant *E. coli* clones positive for pGMV-X were detected by colony PCR using specific primers (Supplementary Table S1). The PCR was performed using Kapa HiFi DNA Polymerase (Kapa Biosystems) according to the manufacturer’s specifications. Amplicons were resolved in 1% agarose gels followed by EtBr staining. Positive clones were purified using the Zymo Miniprep kit (Zymo Research) and sequenced (Macrogen Inc.) to verify correct assembly. Plasmids from correctly assembled pGMV-X *E. coli* clones were electroporated into *Agrobacterium tumefaciens* EHA105 strain as described by Tapia et al. (2009).

**TABLE 1:** Targeted genes in this work and selected guide RNA couples used for their inactivation.

Target gene	Gene ID Phytozome 13*	GenBank accession number**	gRNA1 sequence (5′-3′)***	gRNA2 sequence (5′-3′)***	Expected deletion size (bp)
*VviAIR*12	VIT_200s0184g00110.1	MZ031988	TGTCTGACTTGACTCCGTG	GATTCTCCATCCCCATAGG	878
*VviSWEET*4	VIT_214s0066g01420.1	MZ031989	GACTGGAGCAGACACGGCT	TATCAGATAGAGTCCAGGA	1408
*VviLIN*2	VIT_203s0017g02330.1	MZ031990	AGTCATATCTTGCCACCCA	CCTGCTTACATCTTTGAGG	1360
*VviDEL*1	VIT_217s0000g07630.1	MZ031991	CTTTCTACTGTAAGTGCGA	ATGCAGCAGTCACTAACAG	1427

*According to the genome assembly Genoscope 12X, annotation version 2.1 (Phytozome v13).

**Complete and partial genes cloned from Thompson Seedless cultivar and submitted to GenBank.

***Derived from the “Grapevine CRISPR Search Tool analysis” using scores as computed by Doench et al. (2014) and considering a 19 nucleotide length requirement for cloning, as described in Xing et al. (2014).

### Transient Assay for gRNA Functionality in Grapevine Leaves

A modified protocol from Zottini et al. (2008) was established for use with the one-month potted ‘Thompson Seedless’ plants. Liquid cultures of an *Agrobacterium* clone harboring pGMV-X were grown overnight in 5 mL of Luria-Bertani broth at 28°C with shaking at 200 rpm. *Agrobacterium* infiltration solutions were prepared by diluting aliquots of the overnight culture in 50 mL fresh infiltration medium (50 mM MES, 2 mM Na_3_PO_4_, 0.5% glucose, 100 μM acetosyringone, pH 5.6) and adjusting the OD_600_ to 0.2. These solutions were then incubated in the dark at 25°C for 2 h with gentle agitation (60 rpm). Plant infiltrations were performed by placing 1.5 mL of the *Agrobacterium* infiltration solution (using 3-mL needleless syringes) onto the abaxial face of small-sized leaves (two-thirds of adult size) usually between the third and eighth nodes. Each leaf received 15 infiltrations. The plants were kept under standard growth conditions, and leaf samples were collected between 10 and 14 dpi for genomic DNA (gDNA) extraction.

### Genomic DNA Extraction

Genomic DNA was extracted from grapevine leaves (150 mg/leaf) using the CTAB-based extraction method (Lodhi et al., 1994; Steenkamp et al., 1994) and treated with RNAse A (final concentration 0.1 mg/mL) for 30 min at 37°C.

### Target Editing Identification

Several PCR primers were designed to characterize double gRNA editing sites in the target region; this identified CRISPR/Cas9-induced mutations in both agroinfiltrated grapevine leaves and regenerated plantlets by nested-PCR-based genotyping. Genomic DNA was extracted using the CTAB-based method described above. The DNA concentration was measured using a BioSpec-nano spectrophotometer (Shimadzu Corporation). PCR reactions were performed using Phusion™ High-Fidelity DNA Polymerase (Thermo Scientific™) in a final volume of 25 μL containing 5 μL of 5X Phusion HF buffer, 0.5 μL of 10 mM dNTP mix (Invitrogen™), 0.5 μL of forward primer (10 μM), 0.5 μL of reverse primer (10 μM), 100 ng of gDNA, and 0.2 μL of DNA polymerase (2 U/μL). PCR reactions were performed using the following parameters: 98°C for 30 s, 35 cycles of 98°C for 10 s, 60–64°C (proper annealing temperature was chosen for each gene) for 10 s, and 72°C for 30 or 15 s (for first PCR and second PCR, respectively), and a final extension step of 72°C for 7 min. The products of the first PCR were analyzed by 1.5% agarose gel electrophoresis, and the same amount of PCR products (1/100 dilution; approximately 5 ng/μL) were used as templates for the second PCR (nested-PCR). Amplicons that were positive for GenEd were excised and purified with the Zymoclean Gel DNA Recovery Kit (Zymo Research). The purified DNA fragments were cloned into the pCR™4Blunt-TOPO^®^ vector (Invitrogen™) and sequenced (Macrogen Inc.). For *indel* detection, concentrations of PCR reagents were as described above. Amplifications were carried out using the following profile: 98°C for 30 s, 35 cycles of 98°C for 10 s, annealing at 58–60°C (depending on the gene) for 10 s, and 72°C for 10 s, and a final extension step of 72°C for 7 min. Amplicons were cloned into pCR™4Blunt-TOPO^®^ vector (Invitrogen™) and sequenced (Macrogen Inc). All primers used are listed in Supplementary Table S1.

### Off-Target Analyses

Candidate off-target sequences for selected gRNAs were screened using the “Grapevine CRISPR Search Tool,” and sequences with up to three mismatches in exonic and intronic regions were evaluated. The gRNAs were additionally assessed using the Cas-OFFinder algorithm (Bae et al., 2014) using gRNA1 and gRNA2 for each gene as the query sequences; the number of mismatches were set equal to or less than three and the bulge size was set to one for either the DNA or RNA. Next, primers for the candidate off-target sequences were designed (Supplementary Table S1), and 50 ng of total gDNA from the leaves was used as a template in PCR assays with Kapa HiFi DNA Polymerase (Kapa Biosystems) in accordance with the manufacturer’s instructions. The PCR products were cloned and sequenced for further analysis.

### Release Into Greenhouse and Prospective First Season of Phytopathological Analysis

Acclimatized and conditioned ‘Thompson Seedless’ plants were transferred to the greenhouse after 14–18 months since *Agrobacterium*-mediated gene transfer experiments. Own-rooted ‘Thompson Seedless’ edited plants, including six control wild type (WT) individuals derived from the somatic embryogenesis procedures, were grown in 20-cm diameter bags filled with soil. The plants were arranged in a random block configuration, spaced 15 cm each other and maintained at 20–25°C in greenhouse, watered as required by manual irrigation preventing splash of water onto the leaves. Growth was maintained by exposing the plants to a minimum of 12 h of light/day, using natural light supplemented from March to September with two 1000-watt metal halide lamps. The block was kept under these culturing conditions during the 2020–2021 growing season for development and primary phytopathological evaluation.

### *Erysiphe necator* Assays

Powdery mildew infection is common under greenhouse conditions during the growing season (October to February) in the La Platina Station area, as previously described for other greenhouse studies when no preventive treatment is conducted (Crisp et al., 2008). In a first season under greenhouse conditions, plants were primarily observed for their response to fungal challenges according to material availability. Powdery mildew natural infection in the greenhouse used for the establishment of vines is permanent, and artificial inoculation was not necessary because infection was comprehensive. Vines were arranged in a single block including the 19 double-cut edited vines and six WT (control) individuals randomly allocated. Three fully developed leaves less than eight weeks old were tagged and followed for disease scoring during 14 days since first symptoms detected within the block. Under these conditions, images from three random leaves located between the 5th and 8th nodes were acquired per plant and processed for infection scoring 14 days after first infection signs among individuals in the designed block. The total and infected leaf areas were compared by measuring images with ImageJ 1.49v software (National Institutes of Health, United States), according to considerations described by Pride et al. (2020; visited on 01/03/2021)^3^; each leaf was considered to be an experimental unit (*n* = 3 leaves per plant), and infected areas included sporulation zones, chlorosis, and russet areas. Data was subjected to one-way ANOVA and Fisher’s LSD test (*p* < 0.05) using GraphPad Prism version 8.4.3 software (GraphPad Software, San Diego, CA, United States).

### *Botrytis cinerea* Assays

A virulent isolate of *B. cinerea* was obtained from naturally infected clusters from ‘Thompson Seedless’ plants located in an orchard at La Platina Station (Muñoz et al., 2016). Fungus was prepared and plated on potato dextrose agar at 5°C. An inoculum by fungal growth at 20°C under a diurnal light regime (12/12 light/darkness photoperiod) for 10 days. For harvesting, plates were superficially washed twice with sterile water to extract conidia using a glass rod. Aqueous spore collections were then put into a blender with a few drops of Tween 20. A spore suspension was adjusted (10^7^ conidia/mL) and then transferred into a sterile tube. Three adult leaves (>5th leaf) per individual were used for infections; previously, leaves were cleaned by immersion in sterile water for 30 s, disinfected with 1% (w/v) commercial bleach for 2 min, rinsed twice with water for 2 min, and allowed to dry by placing them on sterile filter papers under a laminar flow hood for 15 min. Dried leaves were discreetly wounded on each lobule and then inoculated with 10 μL of conidial suspension. Inoculated leaves were kept in wet chambers at 17–20°C for 5 days. Control leaves from WT ‘Thompson Seedless’ plants were similarly incubated and inoculated using conidia solutions described above. Infections were scored by measuring necrotic spots (two polar diameters per lesion; Rubio et al., 2015) considering each leaf as an experimental unit. Each infection experiment was in triplicate (*n* = 3 leaves per plant). Data were subjected to one-way ANOVA and Fisher’s LSD test (*p* < 0.05) using GraphPad Prism version 8.4.3 software (GraphPad Software, San Diego, CA, United States).

## Results

### Behavior of Bean Yellow Dwarf Virus-Derived Vector in Grapevine

The ability to replicate LSL vectors in the woody crop *V. vinifera*, a non-natural host for BeYDV, was first evaluated using pLSLGFP.R, a BeYDV-derived vector containing the *GFP* reporter gene. A time course analysis of the GFP fluorescence in both leaves and somatic embryos were performed to determine the replication capacity of the DNA replicons (Figures 1A,B, respectively). Leaves from vacuum-agroinfiltrated plantlets showed an important GFP emission between 5 and 16 dpi (Figures 1A,C), which was maintained as discrete fluorescent spots for up to 23 dpi. Somatic embryos showed an early and strong GFP emission from 4 dpi up to 17 dpi (Figures 1B,D). Discrete GFP spots were also maintained in these tissues up to 33 dpi.

### Assembly of a Universal Geminivirus-Derived Vector for CRISPR/Cas9 Gene Editing

**FIGURE 2:**
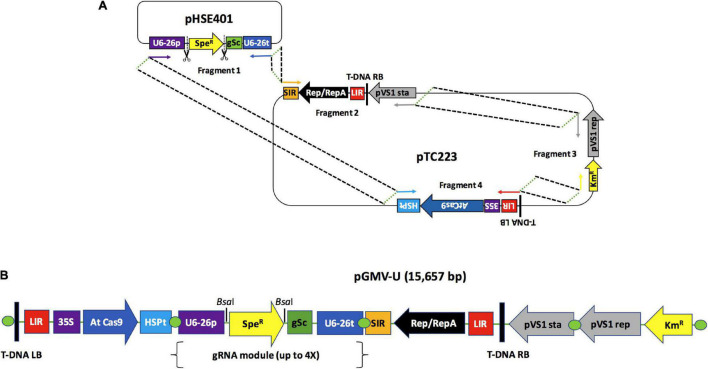
Design and construction of universal geminivirus-based vector for the expression of multiple guide RNAs. **(A)** The geminivirus-based universal vector (pGMV-U) was assembled by linking four DNA fragments through a Gibson assembly reaction. The fragment carrying the sequence required for the insertion of multiple gRNAs was amplified from the pHSE401 plasmid while the three remaining fragments containing sequences for the LSL replicon were located within an *Agrobacterium* T-DNA sequence. The binary vector backbone was amplified from the pTC223 plasmid. Arrows indicate the primers used for the fragment amplifications. Dotted lines indicate the overlapping sequences between fragments required for the assembly reaction. Scissor icons indicate the *Bsa*I sites. **(B)** Resulting pGMV-U vector map: T-DNA RB, right border of the *Agrobacterium* T-DNA; LIR, large intergenic region from the Bean yellow dwarf virus (BeYDV); 35S, 35S *CaMV* promoter from Cauliflower mosaic virus; AtCas9, *Cas*9 *Arabidopsis thaliana* codon usage; HSP t, terminator for heat shock protein 18.2 (HSP) from *A. thaliana*; U6-26p, *A. thaliana* U6-26 RNA *pol*III promoter; Spe*^R^*, spectinomycin resistance gene; gSc, gRNA scaffold sequence; U6-26t, *A. thaliana* U6-26 RNA *pol*III terminator; SIR, short intergenic region from BeYDV; Rep/RepA, nucleotide sequence for the *Rep*/*Rep*A replication genes; LB T-DNA, left border of the *Agrobacterium* T-DNA.

After evaluating the functionality of a BeYDV-derived replicon in grapevine tissues, we next built a universal version of a BeYDV-derived LSL vector by assembling four fragments containing all the important components of a DNA replicon (Figure 2). Figure 2A shows that the donor fragment for a multiple gRNA expression cassette (gSc) corresponds to Fragment 1, a 1964 bp amplicon obtained from the pHSE401 vector. This fragment also included the *At*U6-26 promoter sequence (U6-26p) and the spectinomycin resistance gene (Spe*^R^*) flanked by the *Bsa*I restriction sites required for inserting up to four gRNAs by Golden Gate cloning. The three remaining fragments were obtained as three segments from the plasmid pTC223 and contain the following elements: Fragment 2, a 4050 bp sequence comprising a SIR element, the coding sequence for Rep/RepA, the first LIR, the sequence for the right T-DNA border, and the pVS1sta region. Fragment 3 is a 3872-bp sequence containing the elements necessary for plasmid bacterial replication and a kanamycin resistance gene (Km*^R^*). Fragment 4 is a 5889-bp sequence comprising the sequence for the left T-DNA border, a second LIR element, and the Cas9 expression cassette. These fragments were joined into the final vector by Gibson assembly to generate pGMV-U (Figure 2B), a 15,657-bp vector for plant gene-transfer experiments.

### CRISPR/Cas9-Mediated Gene Editing in Grapevine Using Bean Yellow Dwarf Virus-Derived Replicons

**FIGURE 3:**
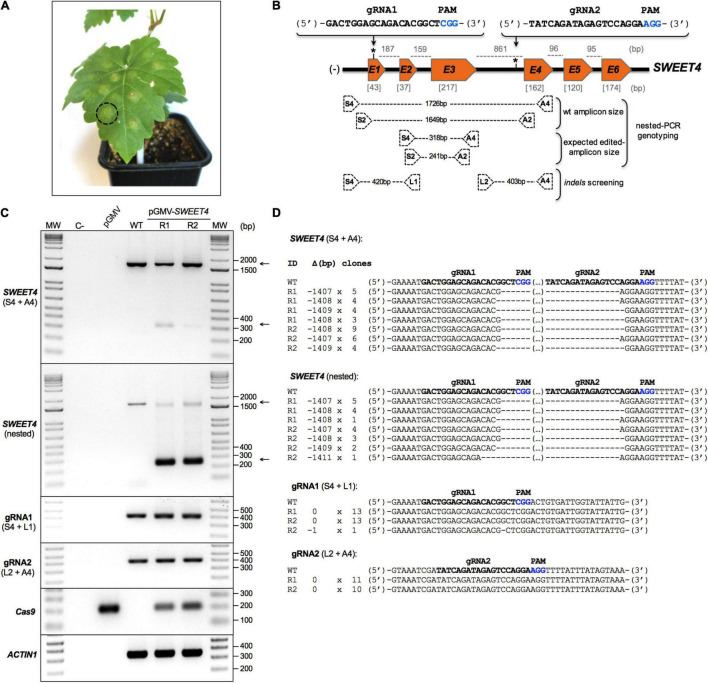
*SWEET4* gene editing in agroinfiltrated ‘Thompson Seedless’ leaves. **(A)** The black dotted circle indicates an agroinfiltration point on a grapevine leaf. **(B)** Schematic illustrating both the mutagenesis target sites in the *SWEET4* gene and nested-PCR-based large deletion detection strategy. Exon (orange boxes) and intron (black line between exons) sizes (bp, base pair) are indicated in gray square brackets and gray dotted lines, respectively. The asterisks indicate the position of the selected gRNA pair for the *SWEET4* gene editing. The gRNAs sequences are shown in bold, and the PAM sequences are highlighted in blue. The location of the primers (black dotted boxes) and the amplicons size (black dotted lines) are shown in a schematic; (–), gene orientation. **(C)** Identification of CRISPR/Cas9-induced mutations in agroinfiltrated grapevine leaves by both direct- and nested-PCR. Genomic DNA from leaf agroinfiltration points was purified and subjected to a first PCR amplification (direct-PCR) using S4 and A4 primers (first upper panel). The PCR products of the primary amplification were then used in a secondary PCR (nested-PCR) using a S2 and A2 primers (second upper panel). The *indel* detection used PCR amplification of gRNA1 (first middle panel) and gRNA2 (second middle panel) target zones using S4 + L1 and L2 + A4 primers, respectively. Arrows (right side of the panel) show the expected edited (lower) and non-edited (upper) versions of the *VviSWEET4* gene. These amplicons were cloned and sequenced for further analysis **(D)**. PCR detection of *Cas9* gene was used to confirm or rule out T-DNA integration (first lower panel). In this case, *Cas9* amplicons were detected in all the agroinfiltrated leaf samples due to a coexistence of both *Agrobacterium* and plant DNAs. *VviACTIN1* was used as a reference gene (second lower panel). All PCR-products were resolved in 1.5% electrophoresis agarose gels. MW, molecular weight marker; C-, negative control (water); pGMV, empty vector control; WT, wild type; R, biological replicate. **(D)** Different types of mutations detected in agroinfiltrated grapevine leaves after CRISPR/Cas9-mediated *SWEET4* genome editing. The columns on the left indicate the sample identification (ID), the deletion size (Δ), and the numbers of clones sequenced (clones). The gRNAs sequences are shown in bold, and the PAM sequences are highlighted in blue.

Gene models for *AIR12*, *SWEET4, LIN2*, and *DEL1* in *Vitis vinifera* were deduced from the ‘Pinot Noir’ reference genome and then experimentally determined in ‘Thompson Seedless’ (Table 1). Based on their gene structure, two different portions of each gene were used in calculations for gRNA design (Supplementary Figure S1A). Upstream and downstream gRNAs for the target region were obtained with strict parameters selected in the “Grapevine CRISPR Search Tool”; these formed candidate gRNA pairs are shown in Supplementary Table S2. A preliminary selection was used for analysis of these elements, and gRNA pairs showing reduced putative off-target activity (see below) and having 40–70% GC content with a preferred C in the variable nucleotide of the PAM were first selected. Preselected pairs per target gene were cloned into pGMV-U (example for *VviDEL1* in Supplementary Figure S1B), and their functionality was evaluated using a fast screening method based on an agroinfiltration assay of young ‘Thompson Seedless’ leaves (Figure 3 and Supplementary Figures S2–S4). The leaf samples were collected between 10 and 14 dpi, and their gDNA was screened for its target gene status (i.e., on-target activity) using a nested-PCR-based genotyping. This strategy increased specificity and sensitivity of the PCR reaction and reduced the false-positive occurrence relying on two sets of amplification primers (*outer* and *inner* primers). Both were located outside of the gRNAs-targeted regions. The results of these PCR-amplifications revealed the presence of both the complete and edited gene versions. Cloning and sequencing these nested-PCR derived amplicons from the different agroinfiltrations showed that they corresponded to deleted versions of each gene (Figure 3 and Supplementary Figures S2–S4) suggesting DNA repairs after CRISPR/Cas9 editing in both ends of these targets.

These assays supported long-term experiments in which ‘Thompson Seedless’ somatic embryos were subjected to gene transfer using each pGMV-X. After approximately 12 months, plantlets were derived from these assays (Supplementary Figure S5A) allowing for leaf sampling and gDNA extraction. Table 2 summarizes the results obtained for these PCR analyses of 473 *in vitro* regenerated plantlets. These materials were rooted, conditioned, and transferred to a greenhouse for whole plant generation after 12 additional months (Supplementary Figure S5B). In a new round of characterization, we next applied new nested-PCR analyses on these plants to confirm a final status for each specific GenEd including elements that could report a transgene status.

**TABLE 2:** Grapevine whole plants derived from editing experiments using pGMV-based vectors.

Target gene	Edited plants/ regenerated plants	Editing rate* (%)	Type of mutant**	Frequency*** (%)
*VviAIR12*	1/32	3.1	chi: 0	0
			Aa: 1	5.3
			aa: 0	0
*VviSWEET4*	6/250	2.4	chi: 1	5.3
			Aa: 5	26.3
			aa: 0	0
*VviLIN2*	6/34	17.6	chi: 2	10.5
			Aa: 4	21.1
			aa: 0	0
*VviDEL1*	6/157	3.8	chi: 0	0
			Aa: 6	31.6
			aa: 0	0

*Editing rates were calculated as edited lines/regenerated plantlets.

**chi, Chimeric; Aa, Heterozygous/Monoallelic; aa, Homozygous or Biallelic.

***The frequency of different mutation types found in the edited lines were calculated as edited lines/total edited lines.

**FIGURE 4:**
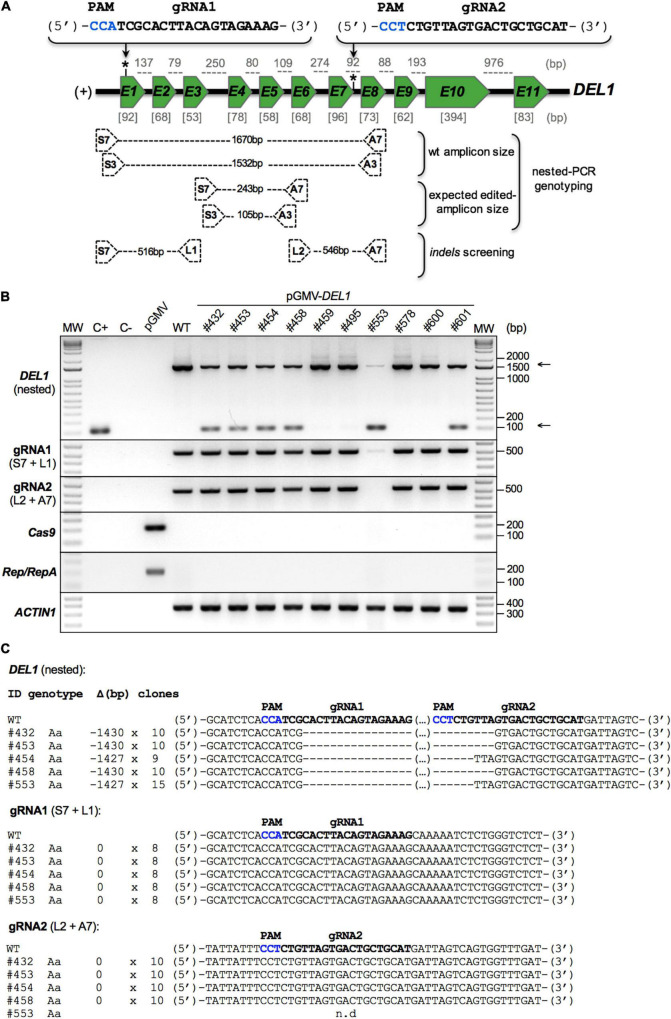
Editing in the *DEL1* gene of ‘Thompson Seedless’ individuals regenerated from somatic embryo gene transfer experiments. Gene transfer experiments were performed in somatic embryos using *Agrobacterium* EHA105 strain harboring the pGMV-*DEL1* vector. After 8 months, whole individuals began to be recovered and developed for further analysis. Genomic DNA was extracted from the leaves of the individuals for use in the *VviDEL1* editing characterization. **(A)** The structure of the gene, the position of the gRNAs (*), and the location of the amplification primers (black dotted boxes) are shown as a schematic. Exon (green boxes) and intron (black line between exons) sizes (bp, base pair) are indicated in gray square brackets and gray dotted lines, respectively. The gRNAs sequences are shown in bold, and the PAM sequences are highlighted in blue; (+), gene orientation. **(B)** Detection of CRISPR/Cas9-induced mutations in regenerated plantlets by nested-PCR. A first PCR amplification was conducted using S7 and A7 primers. The PCR-products of the primary amplification were then used in a secondary PCR (nested-PCR) using a S3 and A3 primers (upper panel). The *indel* detection was carried out by PCR amplification of gRNA1 (first middle panel) and gRNA2 (second middle panel) target zones using S7 + L1 and L2 + A7 primers, respectively. Arrows (right side of the panel) show the expected edited (lower) and non-edited (upper) versions of the *VviDEL1* gene. The presence of two amplification product (upper and lower arrows) suggests a monoallelic/heterozygous or chimeric mutation, while the presence of only one amplicon (lower arrow) suggests a biallelic or homozygous mutations. These amplicons were cloned and sequenced by the Sanger method for further analysis **(C)**. PCR detection of *Cas9* and *Rep/RepA* genes was used to confirm or rule out T-DNA integration (third and fourth middle panel, respectively). *VviACTIN1* was used as a reference gene (lower panel). All PCR-products were resolved in 1.5% electrophoresis agarose gels. MW, molecular weight marker; C+, positive control (S7 + A7 edited amplicon-containing vector); C-, negative control (water); pGMV, empty vector control; WT, wild type; #, line number. **(C)** Edited non-transgenic individuals were sequenced, and different types of mutations were detected in regenerated grapevine plantlets after CRISPR/Cas9-mediated *DEL1* gene editing. The columns on the left indicate the sample identification (ID), the type of mutation (genotype), the deletion size (Δ), and the number of sequenced clones (clones). gRNA sequences are shown in bold, and PAM sequences are highlighted in blue. Aa, Heterozygous/Monoallelic; n.d, non-detected.

Table 2 shows that 19 established plants were considered edited using the double-cut fast screening approach: six for *VviDEL1* (lines #432, #453, #454, #458, #553, #601; gene structure in Figure 4A and mutations in Figure 4B), one for *VviAIR12* (#329; Supplementary Figure S6A), six for *VviSWEET4* (#2, #21, #207, #297. #348, #357; Supplementary Figure S7A), and six for *VviLIN2* (#373, #378, #381, #416, #421, #423; Supplementary Figure S8A). Most of these materials were present in an eventual non-transgenic status as judged by negative *Cas9* and *Rep/RepA* PCR results (Figure 4B, and panel “A” in Supplementary Figures S6–S8), used as marker element for an eventual T-DNA insertion. In addition, targeted double-cut GenEd was also confirmed by cloning and sequencing the PCR products (see Figure 4C and panel “B” in Supplementary Figures S6–S8). We also carried out an *indel* screening in each gRNA-binding site in all double-cut edited lines. No point mutations were found in these lines (exemplified by *DEL1* in Figure 4C).

### Prospective Phytopathological Behavior of Edited Individuals

**FIGURE 5:**
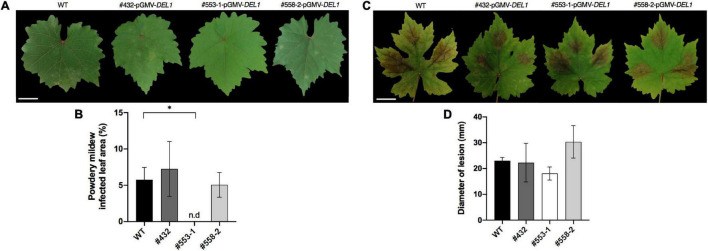
Prospective behavior of edited individuals under fungal challenge. Plants released into the greenhouse were primarily observed for their response to fungal challenge under natural (oidium) and controlled (*Botrytis*) conditions. Vines were arranged in a single block including the 19 double-cut edited vines and six wild type (WT) individuals randomly allocated in the block. Powdery mildew natural infection in this location was established by tagging and scoring of three fully developed leaves per plant over 14 days since first *E. necator* symptoms were first detected **(A)**; responses found in a selected line are then compared **(B)**. Leaf materials from the same individuals in the block were collected and assayed for *B. cinerea* infection using conidia suspensions and controlled fungal growing conditions in humidity chambers in the laboratory **(C)**; and responses found in some events from the group are depicted **(D)**. In this latter, 10 μL aliquots at 10^7^/mL were used, and necrotic lesions were scored 72-h post-inoculation; three biological replicates (*n* = 3 leaves per plant) were performed, and data was subjected to one-way ANOVA and means separated by Fisher’s LSD test (*p* < 0.05) using GraphPad Prism v8.4.3. Asterisk indicates significant differences compared to WT; n.d, non-detected. The white bar represents 3 cm.

During the growing season (October 2020 to February 2021), and under greenhouse conditions in which powdery mildew infection resulted common under no preventive treatments, we calculated the percentage of the leaf surface with visible colonies of *E. necator* (Supplementary Figure S9 and see Supplementary Video). These measurements showed three lines with a higher than 30% reduction in the infection compared to controls (Table 3), highlighting line #553-1 (*VviDEL1*) with over 90% reduction in the observed symptoms (Figures 5A,B).

**TABLE 3:** Powdery mildew infected leaf area rates of representative edited and non-edited grapevine plants under greenhouse conditions.

Target gene	Genotype	Line number	Infected leaf area (%)*		Reduction of infected leaf area (%)**
*VviDEL1*	Aa	553-1	0,0	*	100,0
*VviLIN2*	Aa	378-3	3,4	ns	41,6
*VviDEL1*	Aa	453	3,4	ns	40,6
*VviSWEET4*	AA	393-2	4,5	ns	21,7
*VviSWEET4*	AA	393-1	4,7	ns	18,7
*VviDEL1*	AA	558-2	5,1	ns	12,3
		**WT**	**5,8**		**0,0**
*VviLIN2*	Aa	378-2	6,5	ns	–13,2
*VviDEL1*	Aa	432	7,3	ns	–25,6
*VviLIN2*	Aa	378-1	7,5	ns	–29,5

*Infected leaf area corresponds to affected area mean by powdery mildew compared to total area of three leaves per plant. Image analysis was performed using ImageJ 1.49v software.

**Reduction of infected leaf area corresponds to decrease of the tissue affected by E. necator in percentage compared to WT. Negative values indicate that the symptoms/signs developed were greater than in WT. Values with asterisks indicate significant differences compared with the WT according to Fisher’s LSD test (p < 0.05).

*ns, non-significant. AA, non-edited; Aa, Heterozygous/Monoallelic.*

*Bold values obtained for wild type plants.*

Leaf materials from the same individuals in the block were collected and assayed for *Botrytis cinerea* infection using conidia suspensions and controlled fungal growing conditions using humidity chambers in the laboratory. Three different leaves were scored in these procedures per plant line, and the size of necrotic lesions was scored 72 h post-inoculation (Table 4). In these assays, line #553-1 (*VviDEL1*) reached at least around 20% of size reduction in the necrotic lesions compared to control samples (Figures 5C,D).

**TABLE 4:** Tolerance evaluation against *Botrytis cinerea* of representative edited and non-edited grapevine plants under controlled conditions.

Target gene	Genotype	Line number	Lesion diameter (mm)*		Reduction of lesion (%)**
*VviDEL1*	Aa	553-1	18,09	ns	21,5
*VviLIN2*	Aa	378-2	21,95	ns	4,7
*VviDEL1*	Aa	432	22,29	ns	3,3
*VviLIN2*	Aa	378-1	22,98	ns	0,3
		**WT**	**23,04**		**0,0**
*VviSWEET4*	AA	393-2	24,19	ns	–5,0
*VviDEL1*	AA	558-2	30,34	ns	–31,7
*VviSWEET4*	AA	393-1	30,63	ns	–32,9
*VviAIR12*	AA	365-1	32,03	*	–39,0
*VviDEL1*	Aa	453	34,53	*	–49,9

*3 lobes of a wounded leaf were inoculated with 10 μL of a conidial suspension (10^7^ conidia/mL).

**Lesion reduction corresponds to the decrease of affected tissue by B. cinerea in percentage compared to the lesion developed in WT. Negative values indicate that the developed lesion was greater than in WT. Values with asterisks indicate significant differences compared with the WT according to Fisher’s LSD test (p < 0.05).

*ns, non-significant. AA, non-edited; Aa, Heterozygous/Monoallelic.*

*Bold values obtained for wild type plants.*

## Discussion

Geminivirus-based replicons have been used as tools for heterologous protein expression for more than 30 years (Hayes et al., 1988). Several groups have found that the use of these vectors has definite advantages: they avoid the deleterious effects resulting from having a complete set of viral proteins, they have a large cargo capacity compared to the size limitations seen with whole viruses, and restrictions imposed by the host range may be less stringent (Lozano-Durán, 2016). Several GenEd examples have demonstrated the advantages of these vectors as powerful expression tools for editing proteins and repairing DNA templates (Baltes et al., 2014; Čermák et al., 2015; Butler et al., 2016). In our study, adaptations and improvements were made to the current DNA replicon-based editing strategy, including the construction of a universal LSL vector for expressing the complete CRISPR/Cas9 system components, as well as the incorporation of a multiple gRNA-expression cassette. These cassettes have been identified for the targeted mutation of multiple genes to dissect the functions of gene family members with functional redundancy (Xing et al., 2014). However, some studies indicate that targeted mutations derived from the use of a single gRNA can be repaired by the cell DNA repair machinery. Thus, only two-thirds of the *indels* generated would result in a reading frame shift that affects gene function (Canver et al., 2014). Instead, we pursued a non-reversible large deletion of the selected target genes by removing a considerable amount of their sequences to ensure complete inhibition of function.

Somatic embryogenesis is the most consistent approach for adventitious regeneration, and gene transfer in grapevines has been a continuous focus of development (Tapia et al., 2009; Zhou Q. et al., 2014; Saporta et al., 2016). Recent research has sought to extend the protocols to varieties (Carra et al., 2016; San Pedro et al., 2017), hybrids, and rootstocks (Oláh et al., 2009), including some *Vitis* species, which were until very recently described as recalcitrant (Li et al., 2014). Transgenic individuals were generated using somatic embryo masses (Ren et al., 2016; Nakajima et al., 2017; Wang et al., 2017). In the current study, we used the standard process for gene transfer with pGMV-U. The chimeric or transgenic conditions of the generated individuals cannot be excluded from all results because of the nature of the explant system, and they will need to be characterized extensively. The inclusion of a BeYDV-derived vector expressing GFP in the gene transfer experiments on grapevine somatic embryos in this study resulted in a fundamental milestone. When used in woody fruit species, the results show that the LSL vector behaved similarly to other species and systems (i.e., a 14-dpi time course for evaluable expression; Baltes et al., 2014; Butler et al., 2016; Gil-Humanes et al., 2017). In addition, compared to other gene transfer experiments, no major modifications to the regular embryo regeneration process, including in the time required to generate whole plants, were observed. One disadvantage in our work is the absence of an intermediate selection step during plant regeneration. For this reason, we recently developed a new series of pGMV-U vectors that include reporter genes to visualize the process of selecting the edited explants at the time of transformation. These vectors were used in potatoes (Acha et al., 2021) and have been incorporated into our workflow for GenEd in *Vitis* genotypes.

The inherent technical difficulty of regenerating woody plant species and the time required to generate whole plants reinforces the use of transient expression assays as screening systems prior to molecular breeding (Jelly et al., 2014). Several studies have described the use of cell suspensions, somatic embryos, and protoplasts for transient expression (Jelly et al., 2014). The approach was useful in our primary analysis of the behavior of DNA replicons in grapevine, a non-host species. It showed that the plants infiltrated with a GFP-expressing replicon (pLSLGFP.R) produced the marker protein in the leaves. We propose utilizing these procedures as a testing protocol to probe gRNA cleavage capability within a set of candidate guide molecules before somatic embryo experimentation. This is especially important when a known genetic background is expected to differ from the reference genome used to design the gRNAs, as in this case.

The potential off-target activity of SpCas9 is a concern. Hsu et al. (2013) evaluated SpCas9 cleavage efficiency using gRNAs containing multiple mismatches in human cell lines. These authors reported that gRNAs with up to two mismatches considerably reduced SpCas9 activity, especially if they were in the PAM proximal region. These data suggest that off-target mutations by SpCas9 are rare in plants (Zhang et al., 2014; Wolt et al., 2016). Thus, the “Grapevine CRISPR Search Tool” has been designed to report potential off-target sequence genomes considering up to four possible mismatches between the gRNA and the reference genome sequence (listed in Supplementary Table S3). These predictions showed that gRNA2 addressing the *VviSWEET4* gene had two possible off-targets with three mismatches (one located on an intron on chromosome 11 and the other on an intergenic region on chromosome 15). Although PCR and sequencing confirmed the occurrence of this region in the ‘Thompson Seedless’ genome, we found no major effect of this eventual off-target site in the resulting edited plants (Supplementary Figure S10). These off-target sites were also found by Cas-OFFinder, a processing tool that partially considers the annealing flexibility of possible DNA secondary structures (Bae et al., 2014). Cas-OFFinder reported other potential off-targets for gRNA1 and gRNA2 molecules addressing the *VviSWEET4* gene, but none of these potential sites showed any sequence modification in preliminary experimental analyses. Although these results show the predictive utility of these genome processing tools, they also stress the complexity of this type of analysis.

Pathogen recognition activates signal transduction pathways involving events grouped in the hypersensitivity response (HR), systemic acquired response (SAR), and induced systemic resistance (ISR). The latter was proposed as broad-spectrum defense mechanisms that can act singly or in combination. *Botrytis* has adapted to survive HR, but broad-spectrum responses include important roles for salicylic acid (SA), jasmonic acid (JA), and ethylene. In general, the roles of SA and JA can be split into separate mechanisms that could properly elucidate SA participation in responses against biotrophic pathogens. At the same time, JA can be linked to responses against necrotrophs (Thomma et al., 1998). In this regard, all targeted genes were suggested as potential infection facilitators for several pathogens, including *B. cinerea* and *E. necator*. In the case of *B. cinerea, A. thaliana*, knock-out plants for the orthologous gene *AtSWEET4* confer resistance to this fungus (Chong et al., 2014). In the same study, *VviSWEET4* was strongly up-regulated when challenged with *B. cinerea* and induced plant cell death. Similarly, *A. thaliana AIR12* knock-out plants (a gene encodes a membrane b-type cytochrome) have a strongly decreased susceptibility to this necrotrophic fungus that induces *AtAIR12* expression in susceptible WT plants. In the case of the biotrophic *E. necator*, *AtDEL1* and *AtLIN2* gene mutants associated with SA accumulation can be important factors in disease onset. *Arabidopsis thaliana del1* mutants showed redirected accumulation of SA. Diverting cellular events from development toward plant immunity creates resistance in plants, although only slightly higher than that compared to the WT (Chandran et al., 2014). Similarly, the *A. thaliana* coproporphyrinogen III oxidase LIN2 has been described as a negative regulator of plant defense responses and cell death by decreasing SA levels (Guo et al., 2013). Our preliminary observations, identified line #553-1 as a *VviDEL1* mutant with increased tolerance to natural oidium pressure, in agreement with previous observations made in *Arabidopsis* (Chandran et al., 2014). This is a promising result considering the relevance of *E. necator* and that it is mainly controlled with agrochemicals. Future research will focus on a deeper characterization of this and other lines identified in the current study.

## Conclusion

We designed a straightforward workflow system that led to the establishment of whole grapevine individuals with a targeted gene modified in the first generation. Candidate plants were primarily identified by double-cut gene editing, a property derived from the multi gRNA cloning cassette included in the editing vector. This system avoids transgenic integration, at least based on the current, and uses DNA replicons derived from the geminivirus BeYDV. A prospective analysis of edited plants, based on preliminary phytopathological behavior identified one double-cut *VviDEL1* edited line (#553-1) that was highly tolerant to mildew infection under greenhouse conditions. The results of this work will be subjected to deeper characterization and propagation schedules to define their future potential in our breeding grapevine program.

## Data Availability Statement

The datasets presented in this study can be found in online repositories. The names of the repository/repositories and accession number(s) can be found in the article/Supplementary Material. The “Grapevine CRISPR Search Tool” is available at https://www.fruit-tree-genomics.com, menu Biotools.

## Author Contributions

FO and HP developed gene editing planning. FO, MM, BO, GM, and PP carried out gene transfer and gene editing experimentation. RL and FO carried out the plant characterizations. MM designed and built pGMV-U, also characterized geminivirus replicons in somatic embryos. BO and PP carried out the plant rescuing, maintaining, and propagation. DE carried out the greenhouse plant maintenance. CA developed the “Grapevine CRISPR Search Tool.” RM helped in plant characterization. DR conducted the infection experiments and analyses. HP conceived and organized this research. HP, FO, RV, and RL developed the manuscript. All authors have read and agreed to the published version of the manuscript.

## Conflict of Interest

The authors declare that the research was conducted in the absence of any commercial or financial relationships that could be construed as a potential conflict of interest.

## Publisher’s Note

All claims expressed in this article are solely those of the authors and do not necessarily represent those of their affiliated organizations, or those of the publisher, the editors and the reviewers. Any product that may be evaluated in this article, or claim that may be made by its manufacturer, is not guaranteed or endorsed by the publisher.

## Footnotes

1
snapgene.com

2
http://www.ncbi.nlm.nih.gov/genbank/

3
https://edis.ifas.ufl.edu

## References

[B1] Acha G. Vergara R. Muñoz M. Mora R. Aguirre C. Muñoz M. (2021). A traceable DNA-Replicon derived vector to speed up gene editing in potato: interrupting genes related to undesirable postharvest tuber traits as an example. *Plants* 10:1882. 10.3390/plants10091882 PMC8468489 34579415

[B2] Bae S. Park J. Kim J. S. (2014). Cas-OFFinder: a fast and versatile algorithm that searches for potential off-target sites of Cas9 RNA-guided endonucleases. *Bioinformatics* 30 1473–1475. 10.1093/bioinformatics/btu048 24463181 PMC4016707

[B3] Baker R. F. Leach K. A. Braun D. M. (2012). SWEET as sugar: new sucrose effluxers in plants. *Mol. Plant* 5 766–768. 10.1093/mp/sss054 22815540

[B4] Baltes N. J. Gil-Humanes J. Čermák T. Atkins P. A. Voytas D. F. (2014). DNA replicons for plant genome engineering. *Plant Cell* 26 151–163. 10.1105/tpc.113.119792 24443519 PMC3963565

[B5] Butler N. M. Baltes N. J. Voytas D. F. Douches D. S. (2016). Geminivirus-mediated genome editing in potato (*Solanum tuberosum* L.) using sequence-specific nucleases. *Front. Plant Sci.* 7:1045. 10.3389/fpls.2016.01045 27493650 PMC4955380

[B6] Cai Y. Chen L. Sun S. Wu C. Yao W. Jiang B. (2018). CRISPR/Cas9-mediated deletion of large genomic fragments in soybean. *Int. J. Mol. Sci.* 19:3835. 10.3390/ijms19123835 30513774 PMC6321276

[B7] Canver M. C. Bauer D. E. Dass A. Yien Y. Y. Chung J. Masuda T. (2014). Characterization of genomic deletion efficiency mediated by clustered regularly interspaced palindromic repeats (CRISPR)/Cas9 nuclease system in mammalian cells. *J. Biol. Chem.* 289 21312–21324. 10.1074/jbc.m114.564625 24907273 PMC4118095

[B8] Capriotti L. Baraldi E. Mezzetti B. Limera C. Sabbadini S. (2020). Biotechnological approaches: gene overexpression, gene silencing, and genome editing to control fungal and oomycete diseases in grapevine. *Int. J. Mol. Sci.* 21:5701. 10.3390/ijms21165701 32784854 PMC7460970

[B9] Carra A. Sajeva M. Abbate L. Siragusa M. Pathirana R. Carimi F. (2016). Factors affecting somatic embryogenesis in eight Italian grapevine cultivars and the genetic stability of embryo-derived regenerants as assessed by molecular markers. *Sci. Hortic.* 204 123–127. 10.1016/j.scienta.2016.03.045

[B10] Čermák T. Baltes N. J. Čegan R. Zhang Y. Voytas D. F. (2015). High-frequency, precise modification of the tomato genome. *Genome Biol.* 16 232–245. 10.1186/s13059-015-0796-9 26541286 PMC4635538

[B11] Chandran D. Rickert J. Huang Y. Steinwand M. A. Marr S. K. Wildermuth M. C. (2014). Atypical t2F transcriptional repressor DEL1 acts at the intersection of plant growth and immunity by controlling the hormone salicylic acid. *Cell Host Microbe* 15 506–513. 10.1016/j.chom.2014.03.007 24721578

[B12] Chée R. Pool R. M. (1987). Improved inorganic media constituents for in vitro shoot multiplication of vitis. *Sci. Hortic.* 32 85–95. 10.1016/0304-4238(87)90019-7

[B13] Chialva C. Muñoz C. Miccono M. Eichler E. Calderón L. Prieto H. (2018). Differential expression patterns within the grapevine stilbene synthase gene family revealed through their regulatory regions. *Plant Mol. Biol. Rep.* 36 225–238. 10.1007/s11105-018-1073-3

[B14] Chong J. Piron M. C. Meyer S. Merdinoglu D. Bertsch C. Pere Mestre P. (2014). The SWEET family of sugar transporters in grapevine: VvSWEET4 is involved in the interaction with Botrytis cinerea. *J. Exp. Bot.* 65 6589–6601. 10.1093/jxb/eru375 25246444

[B15] Costa A. Barbaro M. R. Sicilia F. Preger V. Krieger-Liszkay A. Sparla F. (2015). AIR12, a b-type cytochrome of the plasma membrane of *Arabidopsis thaliana* is a negative regulator of resistance against *Botrytis cinerea*. *Plant Sci.* 233 32–43. 10.1016/j.plantsci.2015.01.004 25711811

[B16] Crisp P. Wicks T. J. Lorimer M. Scott E. S. (2008). An evaluation of biological and abiotic controls for grapevine powdery mildew. 1. greenhouse studies. *Aust. J. Grape Wine Res.* 12 192–202. 10.1111/j.1755-0238.2006.tb00059.x

[B17] Di Genova A. Miyasaka A. Muñoz-Espinoza C. Vizoso P. Travisany D. Moraga C. (2014). Whole genome comparison between table and wine grapes reveals a comprehensive catalog of structural variants. *BMC Plant Biol.* 14:7. 10.1186/1471-2229-14-7 24397443 PMC3890619

[B18] Doench J. G. Hartenian E. Graham D. B. Tothova Z. Hegde M. Smith I. (2014). Rational design of highly active sgRNAs for CRISPR-Cas9–mediated gene inactivation. *Nat. Biotechnol.* 32 1262–1267. 10.1038/nbt.3026 25184501 PMC4262738

[B19] Driver J. Kuniyuki A. (1984). In vitro propagation of *Paradox walnut* rootstock. *HortScience* 19 507–509.

[B20] Duan K. Cheng Y. Ji J. Wang C. Wei Y. Wang Y. (2021). Large chromosomal segment deletions by CRISPR/LbCpf1-mediated multiplex gene editing in soybean. *J. Integr. Plant Biol.* 63 1620–1631. 10.1111/jipb.13158 34331750

[B21] Gibson D. G. (2011). Enzymatic assembly of overlapping DNA fragments. *Meth. Enzymol.* 498 349–361. 10.1016/B978-0-12-385120-8.00015-2 21601685 PMC7149801

[B22] Gil-Humanes J. Wang Y. Liang Z. Shan Q. Ozuna C. V. Sánchez-León S. (2017). High-efficiency gene targeting in hexaploid wheat using DNA replicons and CRISPR/Cas9. *Plant J.* 89 1251–1262. 10.1111/tpj.13446 27943461 PMC8439346

[B23] Gomès É Maillot P. Duchêne É (2021). Molecular tools for adapting viticulture to climate change. *Front. Plant Sci.* 12:633846. 10.3389/fpls.2021.633846 33643361 PMC7902699

[B24] Guo C. Wu G. Xing J. Li W. Tang D. Cui B. (2013). A mutation in a coproporphyrinogen III oxidase gene confers growth inhibition, enhanced powdery mildew resistance and powdery mildew-induced cell death in Arabidopsis. *Plant Cell Rep.* 32 687–702. 10.1007/s00299-013-1403-8 23462936

[B25] Hayes R. J. Petty I. T. D. Coutts R. H. A. Buck K. W. (1988). Gene amplification and expression in plants by a replicating geminivirus vector. *Nature* 334 179–182. 10.1038/334179a0

[B26] Hille F. Charpentier E. (2016). CRISPR-Cas: biology, mechanisms? and relevance. *Philos. Trans. R. Soc. B* 371:20150496. 10.1098/rstb.2015.0496 27672148 PMC5052741

[B27] Hsu P. Scott D. Weinstein J. Ran F. A. Konermann S. Agarwala V. (2013). DNA targeting specificity of RNA-guided Cas9 nucleases. *Nat. Biotechnol.* 31 827–832. 10.1038/nbt.2647 23873081 PMC3969858

[B28] Jaillon O. Aury J. M. Noel B. Policriti A. Clepet C. Casagrande A. (2007). The grapevine genome sequence suggests ancestral hexaploidization in major angiosperm phyla. *Nature* 449 463–467. 10.1038/nature06148 17721507

[B29] Jelly N. S. Valat L. Walter B. Maillot P. (2014). Transient expression assays in grapevine: a step towards genetic improvement. *Plant Biotech. J.* 12 1231–1245. 10.1111/pbi.12294 25431200

[B30] Jiang F. Doudna J. A. (2017). CRISPR-Cas9 structures and mechanisms. *Annu. Rev. Biophys.* 46 505–529. 10.1146/annurev-biophys-062215-010822 28375731

[B31] Kapusi E. Corcuera-Gómez M. Melnik S. Stoger E. (2017). Heritable genomic fragment deletions and small indels in the putative ENGase gene induced by CRISPR/Cas9 in barley (2017). *Front. Plant Sci.* 8:540. 10.3389/fpls.2017.00540 28487703 PMC5404177

[B32] Li M.-Y. Jiao Y.-T. Wang Y.-T. Zhang N. Wang B.-B. Liu R.-Q. (2020). CRISPR/Cas9-mediated VvPR4b editing decreases downy mildew resistance in grapevine (*Vitis vinifera* L.). *Hortic Res.* 7:149. 10.1038/s41438-020-00371-4 PMC7458914 32922821

[B33] Li R. Char S. N. Yang B. (2019). Creating large chromosomal deletions in rice using CRISPR/Cas9 (2019). *Meth. Mol. Biol.* 1917 47–61. 10.1007/978-1-4939-8991-1_4 30610627

[B34] Li Z. T. Kim K.-H. Dhekney S. A. Jasinski J. R. Creech M. R. Gray D. J. (2014). An optimized procedure for plant recovery from somatic embryos significantly facilitates the genetic improvement of Vitis. *Hortic. Res.* 1:14027. 10.1038/hortres.2014.27 26504540 PMC4596318

[B35] Li Z. Jayasankar S. Gray D. (2001). Expression of a bifunctional green fluorescent protein (GFP) fusion marker under the control of three constitutive promoters and enhanced derivatives in transgenic grape (*Vitis vinifera*). *Plant Sci.* 160 877–887. 10.1016/s0168-9452(01)00336-3 11297784

[B36] Liu H. Wang K. Jia Z. Gong Q. Lin Z. Du L. (2020). Efficient induction of haploid plants in wheat by editing of TaMTL using an optimized agrobacterium-mediated CRISPR system. *J. Exp. Bot.* 71 1337–1349. 10.1093/jxb/erz529 31760434 PMC7031065

[B37] Lodhi M. A. Ye G. N. Weeden N. F. Reisch B. I. (1994). A simple and efficient method for DNA extraction from grapevine cultivars, Vitis species and Ampelopsis. *Plant Mol. Biol. Rep.* 12 6–13.

[B38] Lozano-Durán R. (2016). Geminiviruses for biotechnology: the art of parasite taming. *New Phytol.* 210 58–64. 10.1111/nph.13564 26214399

[B39] Malnoy M. Viola R. Jung M.-H. Koo O.-J. Kim S. Kim J.-S. (2016). DNA-free genetically edited grapevine and apple protoplast using CRISPR/Cas9 ribonucleoproteins. *Front. Plant Sci.* 7:1904. 10.3389/fpls.2016.01904 28066464 PMC5170842

[B40] Moriondo M. Bindi M. Fagarazzi C. Ferrise R. Trombi G. (2011). Framework for high-resolution climate change impact assessment on grapevines at a regional scale. *Reg. Environ. Change* 11 553–567. 10.1007/s10113-010-0171-z

[B41] Muñoz G. Camposa F. Salgado D. Galdames R. Gilchrist L. Chahin G. (2016). Molecular identification of *Botrytis cinerea*, Botrytis paeoniae and Botrytis pseudocinerea associated with gray mould disease in peonies (i Pall.) in Southern Chile. *Rev. Iberoam. Micol.* 33 43–47. 10.1016/j.riam.2015.02.002 25982419

[B42] Murashige T. Skoog F. (1962). A revised medium for rapid growth and bioassays with tobacco tissue cultures. *Physiol. Plant.* 15 473–497. 10.1111/j.1399-3054.1962.tb08052.x

[B43] Nakajima I. Ban Y. Azuma A. Onoue N. Moriguchi T. Yamamoto T. (2017). CRISPR/Cas9-mediated targeted mutagenesis in grape. *PLoS One* 12:e0177966. 10.1371/journal.pone.0177966 28542349 PMC5436839

[B44] Nitsch J. P. Nitsch C. (1969). Haploid plants from pollen grains. *Science* 163 85–87. 17780179 10.1126/science.163.3862.85

[B45] Oh Y. Kim H. Kim S.-G. (2021). Virus-induced plant genome editing. *Curr. Opin. Plant Biol.* 60:101992. 10.1016/j.pbi.2020.101992 33450609

[B46] Oláh R. Zok A. Pedryc A. Howard S. Kovács L. G. (2009). Somatic embryogenesis in a broad spectrum of grape genotypes. *Sci. Hortic.* 120 134–137.

[B47] Paul N. C. Park S.-W. Liu H. Choi S. Ma J. MacCready J. S. (2021). Plant and fungal genome editing to enhance plant disease resistance using the CRISPR/Cas9 system. *Front. Plant Sci.* 12:700925. 10.3389/fpls.2021.700925 34447401 PMC8382960

[B48] Pride L. Vallad G. Agehara S. (2020). *How to Measure Leaf Disease Damage Using Image Analysis in ImageJ.* Florida: UF/IFAS.

[B49] Pulido-Quetglas C. Aparicio-Prat E. Arnan C. Polidori T. Hermoso T. Palumbo E. (2017). Scalable design of paired CRISPR guide RNAs for genomic deletion. *PLoS Comput. Biol.* 13:e1005341. 10.1371/journal.pcbi.1005341 28253259 PMC5333799

[B50] Ren C. Liu X. Zhang Z. Wang Y. Duan W. Liang Z. (2016). CRISPR/Cas9-mediated efficient targeted mutagenesis in Chardonnay (*Vitis vinifera* L.). *Sci. Rep.* 6:32289. 10.1038/srep32289 27576893 PMC5006071

[B51] Rubio J. Montes C. Castro Á Álvarez C. Olmedo B. Muñoz M. (2015). Genetically engineered thompson seedless grapevine plants designed for fungal tolerance: selection and characterization of the best performing individuals in a field trial. *Trans. Res.* 24 43–60. 10.1007/s11248-014-9811-2 25011563

[B52] San Pedro T. Gammoudi N. Peiró R. Olmos A. Gisbert C. (2017). Somatic embryogenesis from seeds in a broad range of *Vitis vinifera* L. varieties: rescue of true-to-type virus-free plants. *BMC Plant Biol.* 17:226. 10.1186/s12870-017-1159-3 29187140 PMC5706158

[B53] Saporta R. San Pedro T. Gisbert C. (2016). Attempts at grapevine (*Vitis vinifera* L.) breeding through genetic transformation: the main limiting factors. *Vitis* 55 173–186.

[B54] Scintilla S. Salvagnin U. Giacomelli L. Zeilmaker T. Malnoy M. A. van der Voort J. R. (2021). Regeneration of plants from DNA-free edited grapevine protoplasts. *BioRxiv.* Available Online at: 10.1101/2021.07.16.452503. (accessed July 20, 2021). PMC9752144 36531381

[B55] Shen B. Zhang W. Zhang J. Zhou J. Wang J. Chen L. (2014). Efficient genome modification by CRISPR-Cas9 nickase with minimal off-target effects. *Nat. Methods* 11 399–402. 10.1038/nmeth.2857 24584192

[B56] Steenkamp J. Wild I. Lourens A. Van Helden P. (1994). Improved method for DNA extraction from *Vitis vinifera*. *Am. J. Enol. Vitic.* 45 102–106.

[B57] Sternberg S. H. Redding S. Jinek M. Greene E. C. Doudna J. A. (2014). DNA interrogation by the CRISPR RNA-guided endonuclease Cas9. *Nature* 507 62–67. 10.1038/nature13011 24476820 PMC4106473

[B58] Tapia E. Sequeida Á Castro Á Montes C. Zamora P. López R. (2009). Development of grapevine somatic embryogenesis using an air-lift bioreactor as an efficient tool in the generation of transgenic plants. *J. Biotechnol.* 139 95–101. 10.1016/j.jbiotec.2008.09.009 18984020

[B59] Thomma B. Eggermont K. Penninckx I. Mauch-Mani B. Vogelsang R. Cammue B. (1998). Separate jasmonate-dependent and salicylate-dependent defense-response pathways in Arabidopsis are essential for resistance to distinct microbial pathogens. *Proc. Natl. Acad. Sci.* 95 15107–15111. 10.1073/pnas.95.25.15107 9844023 PMC24583

[B60] van der Oost J. Westra E. R. Jackson R. N. Wiedenheft B. (2014). Unravelling the structural and mechanistic basis of CRISPR–Cas systems. *Nat. Rev. Microbiol.* 12 479–492. 10.1038/nrmicro3279 24909109 PMC4225775

[B61] Vergara R. Olivares F. Olmedo B. Toro C. Muñoz M. Zúñiga C. (2021). “Gene editing in Prunus spp.: the challenge of adapting regular gene transfer procedures for precision breeding,” in *Prunus - Recent Advances*, ed. Küden A. (London: IntechOpen).

[B62] Wang X. Tu M. Wang D. Liu J. Li Y. Li Z. (2018). CRISPR/Cas9-mediated efficient targeted mutagenesis in grape in the first generation. *Plant Biotechnol. J.* 16 844–855. 28905515 10.1111/pbi.12832 PMC5866948

[B63] Wang Y. Geng L. Yuan M. Wei J. Jin C. Li M. (2017). Deletion of a target gene in Indica rice via CRISPR/Cas9. *Plant Cell Rep.* 36 1333–1343. 10.1007/s00299-017-2158-4 28584922

[B64] Wildermuth M. C. (2010). Modulation of host nuclear ploidy: a common plant biotroph mechanism. *Curr. Opin. Plant Biol.* 13 449–458. 10.1016/j.pbi.2010.05.005 20542725

[B65] Wolt J. D. Wang K. Sashital D. Lawrence-Dill C. J. (2016). Achieving plant CRISPR targeting that limits off-target effects. *Plant Genome* 9 1–8. 10.3835/plantgenome2016.05.0047 27902801

[B66] Wu R. Lucke M. Jang Y. T. Zhu W. Symeonidi E. Wang C. (2018). An efficient CRISPR vector toolbox for engineering large deletions in *Arabidopsis thaliana*. *Plant Methods* 14:65. 10.1186/s13007-018-0330-7 30083222 PMC6071326

[B67] Xing H. L. Dong L. Wang Z. P. Zhang H. Y. Han C. Y. Liu B. (2014). A CRISPR/Cas9 toolkit for multiplex genome editing in plants. *BMC Plant Biol.* 14:327. 10.1186/s12870-014-0327-y 25432517 PMC4262988

[B68] Zhang H. Zhang J. Wei P. Zhang B. Gou F. Feng Z. (2014). The CRISPR/Cas9 system produces specific and homozygous targeted gene editing in rice in one generation. *Plant Biotechnol. J.* 12 97–807. 10.1111/pbi.12200 24854982

[B69] Zhao Y. Zhang C. Liu W. Gao W. Liu C. Song G. (2016). An alternative strategy for targeted gene replacement in plants using a dual-sgRNA/Cas9 design. *Sci. Rep.* 6:23890. 10.1038/srep23890 27033976 PMC4817149

[B70] Zhou H. Liu B. Weeks D. P. Spalding M. H. Yang B. (2014). Large chromosomal deletions and heritable small genetic changes induced by CRISPR/Cas9 in rice. *Nucleic Acids Res.* 42 10903–10914. 10.1093/nar/gku806 25200087 PMC4176183

[B71] Zhou Q. Dai L. Cheng S. He J. Wang D. Zhang J. (2014). A circulatory system useful both for long-term somatic embryogenesis and genetic transformation in Vitis vinifera L. cv. ‘Thompson Seedless’. *Plant Cell Tissue Org. Cult.* 118 157–168. 10.1007/s11240-014-0471-y

[B72] Zhu H. Li C. Gao C. (2020). Applications of CRISPR/Cas in agriculture and plant biotechnology. *Nat. Rev. Mol. Cell Biol.* 21 661–677. 10.1038/s41580-020-00288-9 32973356

[B73] Zottini M. Barizza E. Costa A. Formentin E. Ruberti C. Carimi F. (2008). Agroinfiltration of grapevine leaves for fast transient assays of gene expression and for long-term production of stable transformed cells. *Plant Cell Rep.* 27 845–853. 10.1007/s00299-008-0510-4 18256839

