# Supplementary material for: CRISPR/Cas9 Targeted Editing of Genes Associated With Fungal Susceptibility in Vitis vinifera L. cv. Thompson Seedless Using Geminivirus-Derived Replicons
Source: Front Plant Sci. 2021 Dec 23;12:791030. doi: 10.3389/fpls.2021.791030 (PMC8733719; doi:10.3389/fpls.2021.791030)
Supplement: Supplementary file 1 [file Data_Sheet_1.docx]

Supplementary Material

# Supplementary Tables

**Supplementary Table S1.** List of primers used in this work.

**Supplementary Table S2.** Guide RNAs designed for ‘susceptibility trait’ gene editing.

**Supplementary Table S3.** Off-targets defined by the ‘Grapevine CRISPR Search Tool’.

# Supplementary Figures


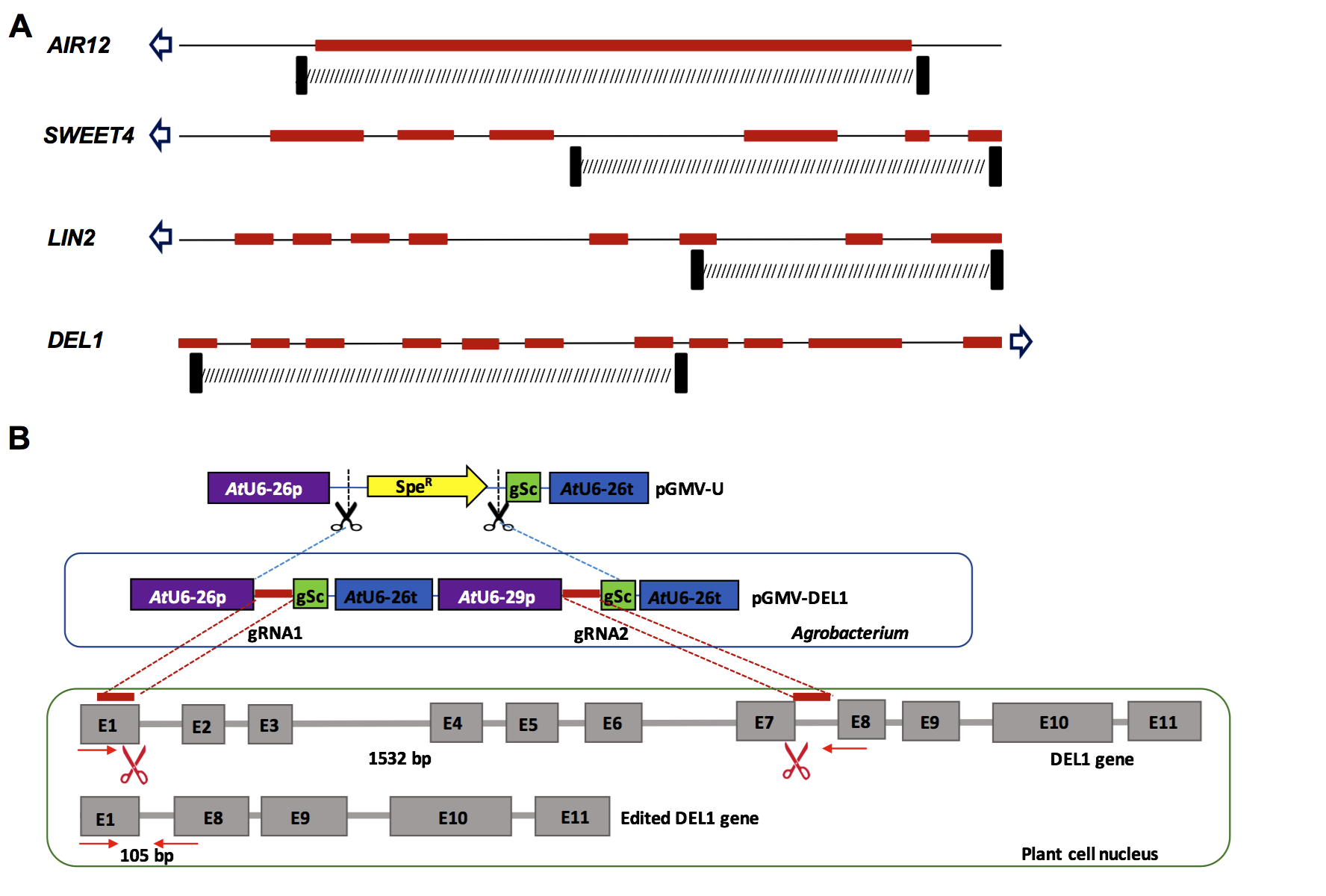


**Supplementary Figure S1.** **Target gene structures and expected deletions by double-cut editing.** Genes eventually associated to pathogen susceptibility were selected in this study. Diagrams showing structures for the *Vitis vinifera* genes *AUXIN INDUCED IN ROOT CULTURE* (*AIR12*), *SUGARS WILL EVENTUALLY BE EXPORTED TRANSPORTER 4* (*SWEET4*), the *LESION INITIATION 2* (*LIN2*), and the *DIMERIZATION PARTNER E2F LIKE 1* (*DEL1*) are shown. Exons, red boxes; introns, black line between exons; arrows, gene orientation **(A)**. The corresponding genomic sequences (gene locations in Table 1) and structures were established in the reference genome and processed by the ‘Grapevine CRISPR Search Tool’. The target zones for gene editing (dashed boxes) were defined, leading the search tool to adjust the gRNA target zones upstream and downstream in a process running under ‘strict search’ parameters. From this analysis, a list of gRNA pairs was obtained, and the best gRNA pairs selected (black boxes) and assembled in particular versions in pGMV-U. Assembly of the gRNA pairs into pGMV-U were carried out by Golden Gate reaction of each independent gRNA module into the gRNA Scaffold (gSc) of the vector, generating a predicted editing of each target gene, as exemplified for *VviDEL1* gene **(B)**.


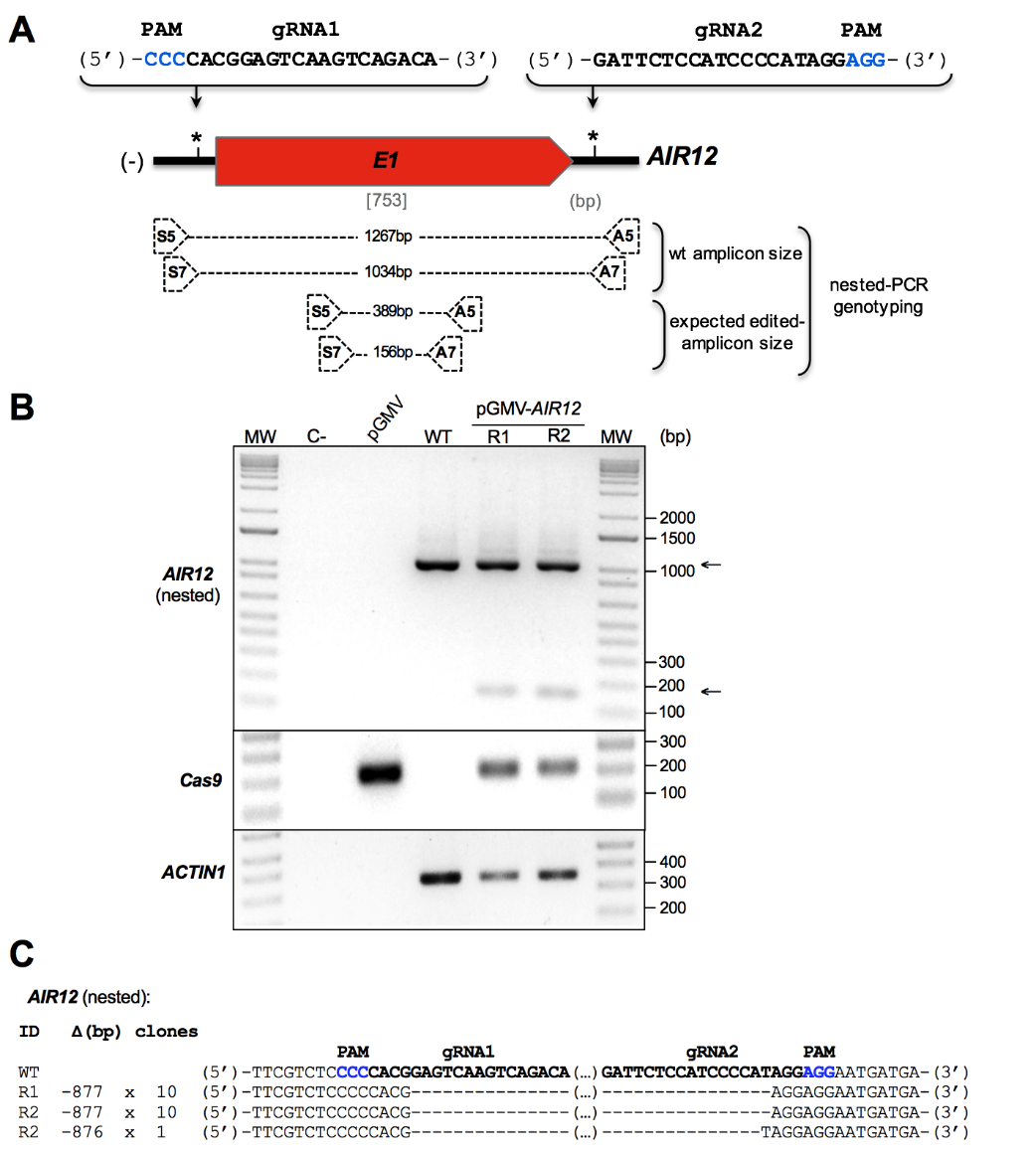


**Supplementary Figure S2.** ***AIR12* gene editing in agroinfiltrated ‘Thompson Seedless’ leaves.** **(A)** The structure of the gene, the position of the gRNAs and the location of the amplification primers are schematized. **(B)** Identification of CRISPR/Cas9-induced mutations in agroinfiltrated grapevine leaves by nested-PCR (for more details, see figure legend 3). MW, molecular weight marker; C-, negative control (water); pGMV, empty vector control; WT, wild type; R, biological replicate. **(C)** Different types of mutations detected in agroinfiltrated grapevine leaves after CRISPR/Cas9-mediated *AIR12* gene editing. The columns on the left indicate the sample identification (ID), the deletion size (**∆**), and the number of clones sequenced (clones). gRNA sequences are shown in bold and PAM sequences are highlighted in blue.


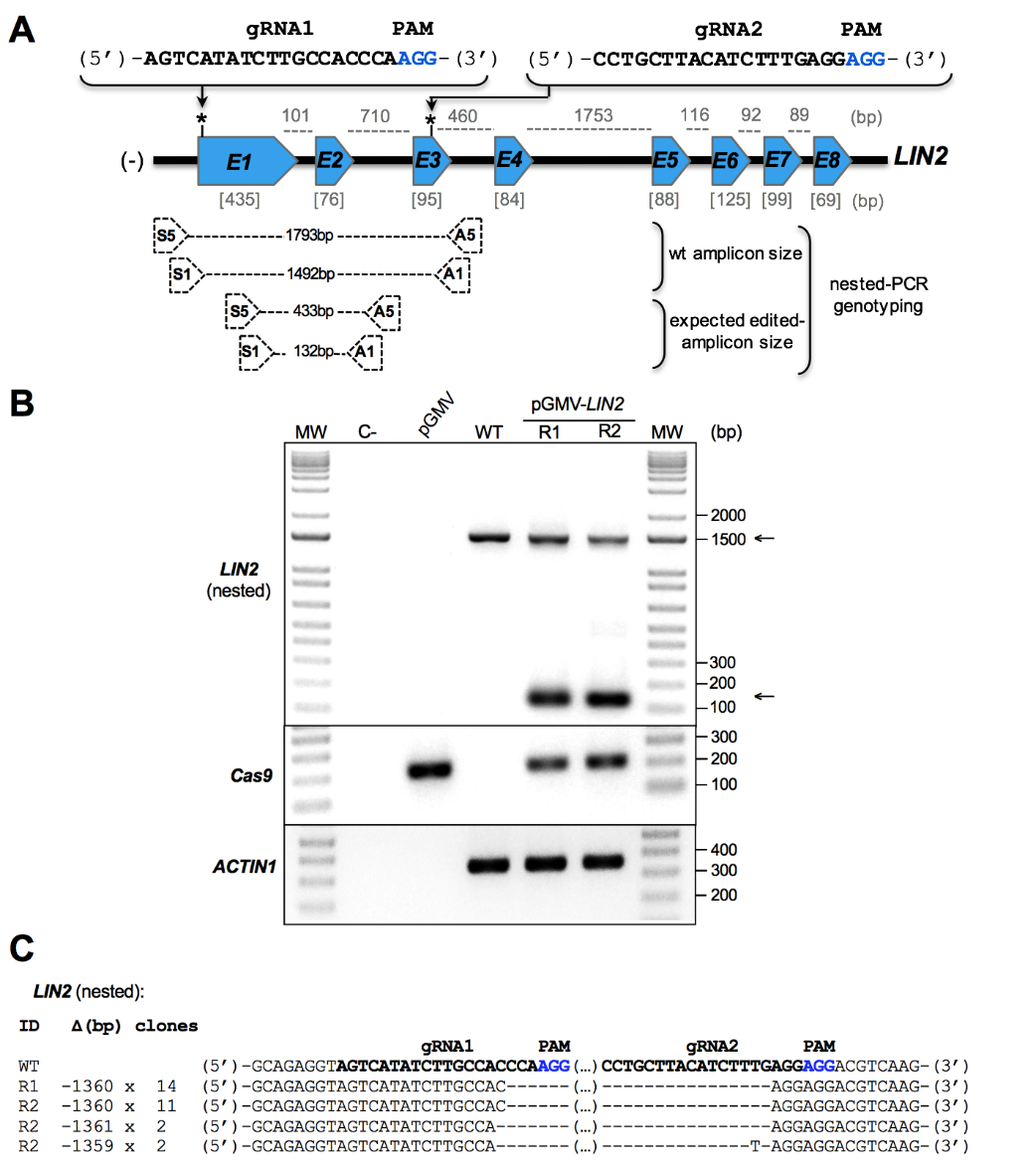


**Supplementary Figure S3.** ***LIN2* gene editing in agroinfiltrated ‘Thompson Seedless’ leaves.** **(A)** The structure of the gene, the position of the gRNAs and the location of the amplification primers are schematized. **(B)** Identification of CRISPR/Cas9-induced mutations in agroinfiltrated grapevine leaves by nested-PCR (for more details, see figure legend 3). MW, molecular weight marker; C-, negative control (water); pGMV, empty vector control; WT, wild type; R, biological replicate. **(C)** Different types of mutations detected in agroinfiltrated grapevine leaves after CRISPR/Cas9-mediated *LIN2* gene editing. The columns on the left indicate the sample identification (ID), the deletion size (**∆**), and the number of clones sequenced (clones). gRNA sequences are shown in bold and PAM sequences are highlighted in blue.


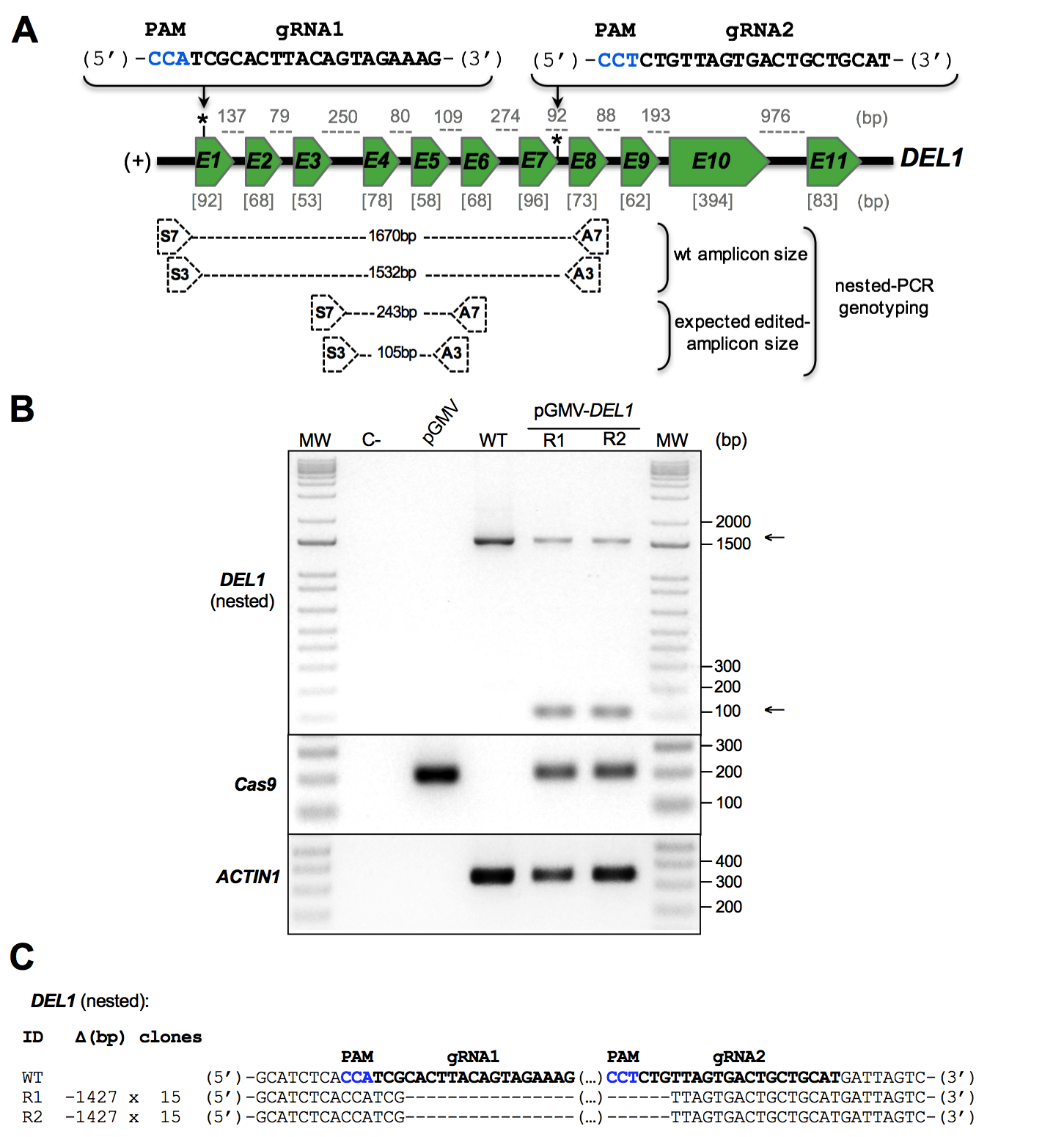


**Supplementary Figure S4.** ***DEL1* gene editing in agroinfiltrated ‘Thompson Seedless’ leaves.** **(A)** The structure of the gene, the position of the gRNAs and the location of the amplification primers are schematized. **(B)** Identification of CRISPR/Cas9-induced mutations in agroinfiltrated grapevine leaves by nested-PCR. (for more details, see figure legend 3). MW, molecular weight marker; C-, negative control (water); pGMV, empty vector control; WT, wild type; R, biological replicate. **(C)** Different types of mutations detected in agroinfiltrated grapevine leaves after CRISPR/Cas9-mediated *DEL1* gene editing. The columns on the left indicate the sample identification (ID), the deletion size (**∆**), and the number of clones sequenced (clones). gRNA sequences are shown in bold and PAM sequences are highlighted in blue.


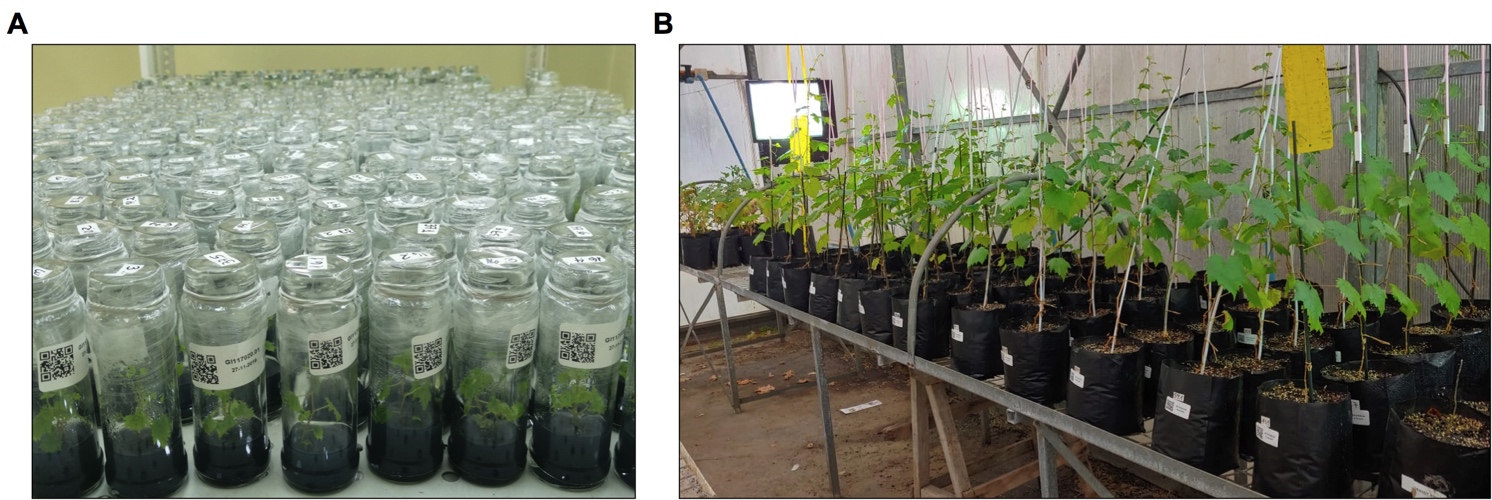


**Supplementary Figure S5.** **Plantlets derived from gene transfer assays including gene editing process.** The *in vitro* stage **(A)** and the greenhouse release **(B)** after a total time of approximately 24 months.


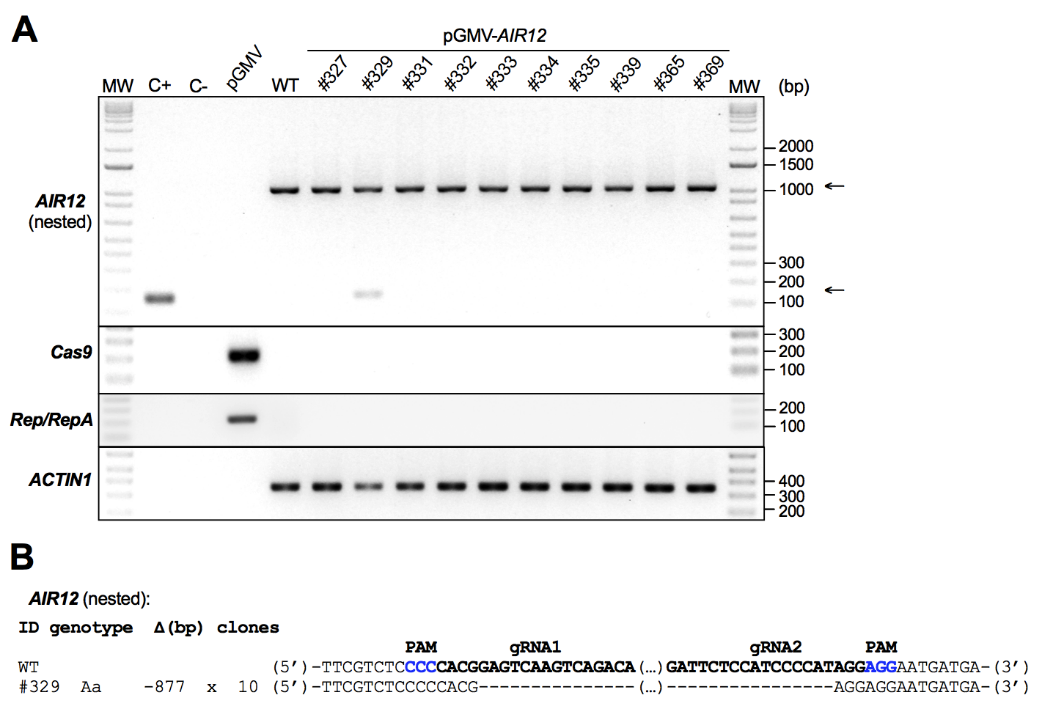


**Supplementary Figure S6.** **Editing in the *AIR12* gene of ‘Thompson Seedless’ individuals regenerated from somatic embryo gene transfer experiments.** Gene transfer experiments were performed in somatic embryos using *Agrobacterium* EHA105 strain harboring the pGMV-*AIR12* vector. **(A)** Detection of CRISPR/Cas9-induced mutations in regenerated plantlets by nested-PCR (for more details, see figure legend 4). MW, molecular weight marker; C+, positive control (S5 + A5 edited amplicon-containing vector); C-, negative control (water); pGMV, empty vector control; WT, wild type; #, line number. **(B)** Edited non-transgenic individuals were sequenced and different types of mutations were detected in regenerated grapevine plantlets after CRISPR/Cas9-mediated *AIR12* gene editing. The columns on the left indicate the sample identification (ID), the type of mutation (genotype), the deletion size (**∆**), and the numbers of clones sequenced (clones). gRNA sequences are shown in bold and PAM sequences are highlighted in blue. chi., chimeric; Aa, monoallelic/heterozygous.


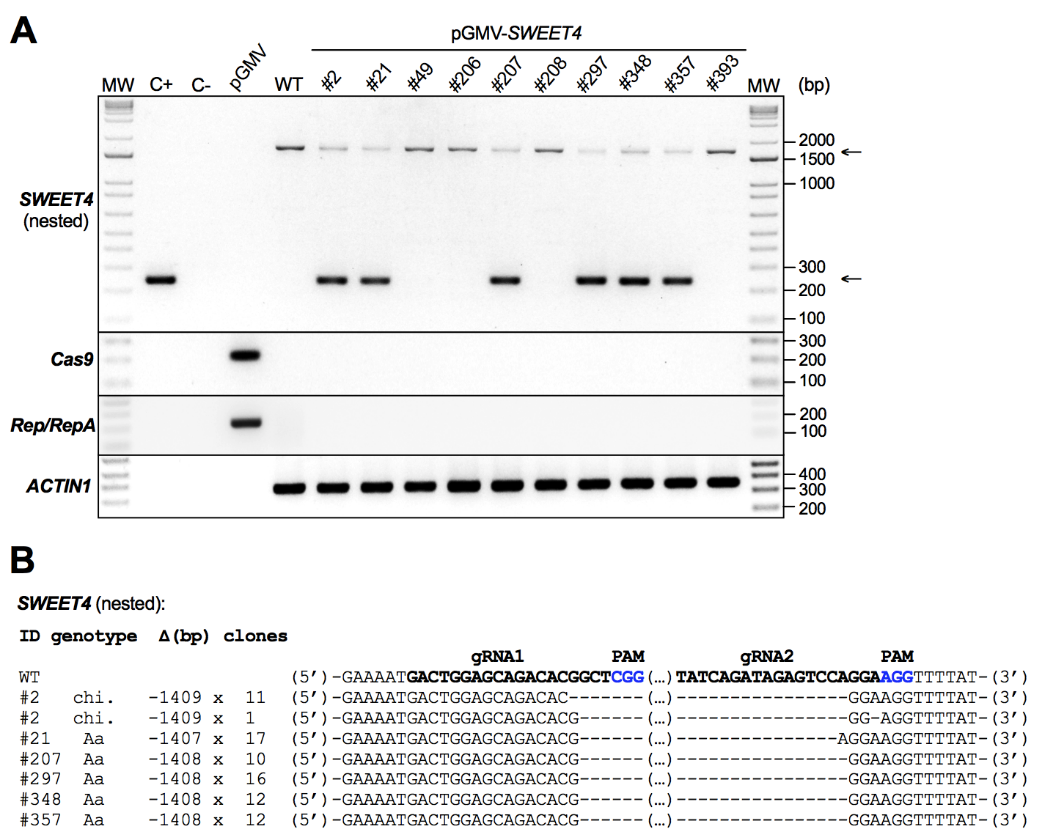


**Supplementary Figure S7.** **Editing in the *SWEET4* gene of ‘Thompson Seedless’ individuals regenerated from somatic embryo gene transfer experiments.** Gene transfer experiments were performed in somatic embryos using *Agrobacterium* EHA105 strain harboring the pGMV-*SWEET4* vector. **(A)** Detection of CRISPR/Cas9-induced mutations in regenerated plantlets by nested-PCR (for more details, see figure legend 4). MW, molecular weight marker; C+, positive control (S4 + A4 edited amplicon-containing vector); C-, negative control (water); pGMV, empty vector control; WT, wild type; #, line number. **(B)** Edited non-transgenic individuals were sequenced and different types of mutations were detected in regenerated grapevine plantlets after CRISPR/Cas9-mediated *SWEET4* gene editing. The columns on the left indicate the sample identification (ID), the type of mutation (genotype), the deletion size (**∆**), and the numbers of clones sequenced (clones). gRNA sequences are shown in bold and PAM sequences are highlighted in blue. chi., chimeric; Aa, monoallelic/heterozygous.


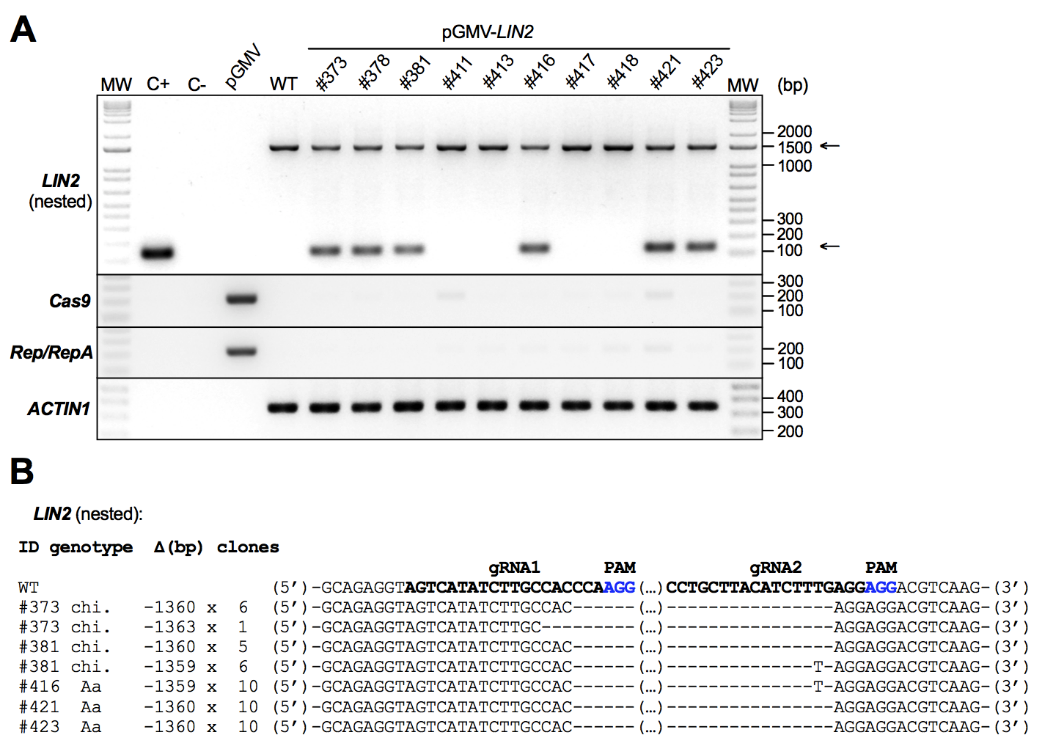


**Supplementary Figure S8.** **Editing in the *LIN2* gene of ‘Thompson Seedless’ individuals regenerated from somatic embryo gene transfer experiments.** Gene transfer experiments were performed in somatic embryos using *Agrobacterium* EHA105 strain harboring the pGMV-*LIN2* vector. **(A)** Detection of CRISPR/Cas9-induced mutations in regenerated plantlets by nested-PCR (for more details, see figure legend 4). MW, molecular weight marker; C+, positive control (S5 + A5 edited amplicon-containing vector); C-, negative control (water); pGMV, empty vector control; WT, wild type; #, line number. **(B)** Edited non-transgenic individuals were sequenced and different types of mutations were detected in regenerated grapevine plantlets after CRISPR/Cas9-mediated *LIN2* gene editing. The columns on the left indicate the sample identification (ID), the type of mutation (genotype), the deletion size (**∆**), and the numbers of clones sequenced (clones). gRNA sequences are shown in bold and PAM sequences are highlighted in blue. chi., chimeric; Aa, monoallelic/heterozygous.


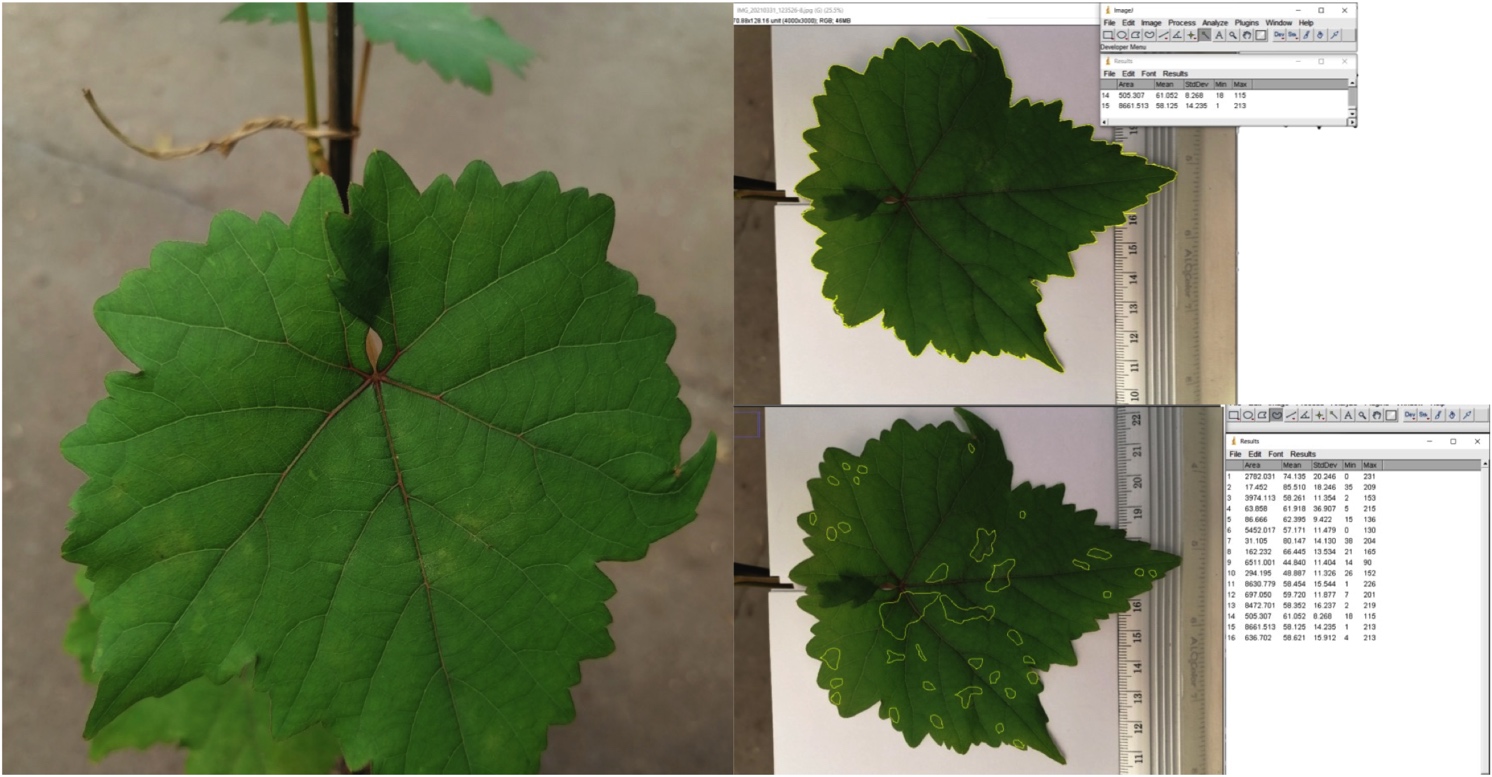


**Supplementary Figure S9.** **Calculation of powdery mildew infection.** Infected and total leaf areas were compared by processing digital images acquired from the tagged leaves in the edited greenhouse plants (left) using the ImageJ software as shown at right. Yellow lines show drafted areas for total (upper right) and infected (lower right) zones identified on each sample.


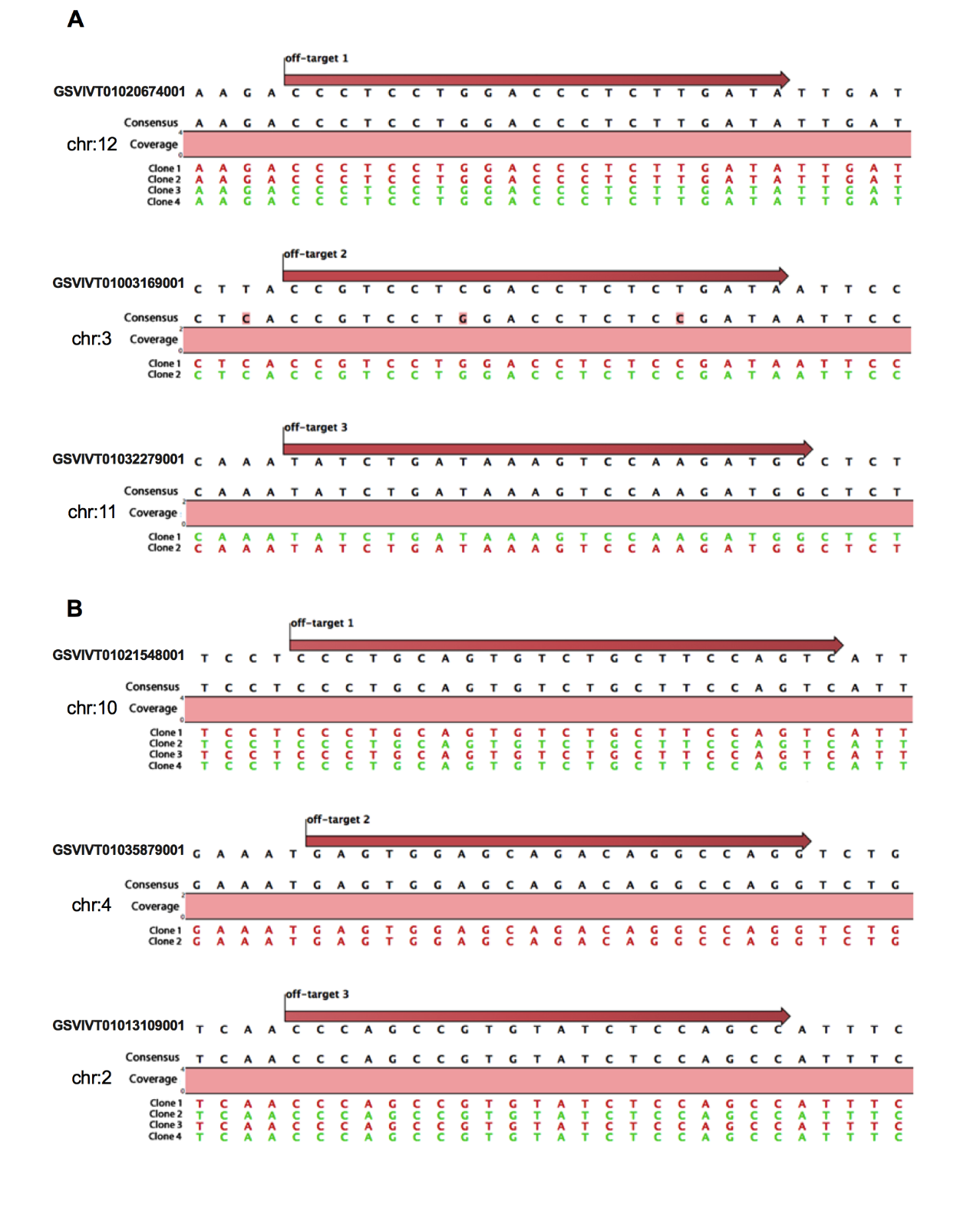


**Supplementary Figure S10.** **Off-target activity of the gRNA pairs used for editing the *SWEET4* gene in ‘Thompson Seedless’ grapevine.** Off-target locations were determined using the ‘Grapevine CRISPR Search Tool’ (Supplementary Table S3). The only result obtained for an intragenic off-target region, predicted for one of the gRNAs targeting the *VviSWEET4 gene* (i.e., gRNA2 chr11) was experimentally checked. PCR primers were designed (Supplementary Table S1) and amplifications were performed and resolved by agarose gel electrophoresis. In this particular case, results from CasOffinder predicted other off-target events which were also reviewed for eventual gRNA activity; analyses including the gRNA2 sites at chr12, and chr3 **(A)** and for gRNA1 at chr10, chr4, and chr2 **(B)** are shown. Arrows indicate the relevant amplification products for the unedited off-target reference sequence.
